# An Extensive Network of Information Flow through the B1b/c Intersubunit Bridge of the Yeast Ribosome

**DOI:** 10.1371/journal.pone.0020048

**Published:** 2011-05-19

**Authors:** Michael H. J. Rhodin, Jonathan D. Dinman

**Affiliations:** Department of Cell Biology and Molecular Genetics, University of Maryland, College Park, Maryland, United States of America; Texas A&M University, United States of America

## Abstract

Yeast ribosomal proteins L11 and S18 form a dynamic intersubunit interaction called the B1b/c bridge. Recent high resolution images of the ribosome have enabled targeting of specific residues in this bridge to address how distantly separated regions within the large and small subunits of the ribosome communicate with each other. Mutations were generated in the L11 side of the B1b/c bridge with a particular focus on disrupting the opposing charge motifs that have previously been proposed to be involved in subunit ratcheting. Mutants had wide-ranging effects on cellular viability and translational fidelity, with the most pronounced phenotypes corresponding to amino acid changes resulting in alterations of local charge properties. Chemical protection studies of selected mutants revealed rRNA structural changes in both the large and small subunits. In the large subunit rRNA, structural changes mapped to Helices 39, 80, 82, 83, 84, and the peptidyltransferase center. In the small subunit rRNA, structural changes were identified in helices 30 and 42, located between S18 and the decoding center. The rRNA structural changes correlated with charge-specific alterations to the L11 side of the B1b/c bridge. These analyses underscore the importance of the opposing charge mechanism in mediating B1b/c bridge interactions and suggest an extensive network of information exchange between distinct regions of the large and small subunits.

## Introduction

The ribosome must coordinate a series of rapid, highly accurate events between multiple locations and molecular complexes through every stage of protein synthesis. During the elongation cycle alone, these include discriminating between cognate and non- or near-cognate aminoacyl-tRNA (aa-tRNA) complexed with elongation factor eEF1A (EF-Tu in bacteria and archaea), accommodating aa-tRNAs into the peptidyltransferase center (PTC) of the large subunit (LSU), catalyzing peptide bond formation, and recruiting and stimulating GTP hydrolysis by eEF2 (EF-G in bacteria and archaea). The cycle is completed by translocation of the ribosome along the mRNA by three nucleotides in the 3′ direction. While our understanding of the biochemistry and local structural changes that occur during each step of elongation are becoming clear, deciphering how numerous widely separated regions of this macromolecular complex coordinate their actions with one another is less apparent. Mapping of the allosteric communication pathways between different functional centers to better understand how the ribosome synchronizes the various steps of translation, particularly between the large and small subunits, is thus a critical challenge to understanding the relationship between ribosome structure and function.

**Figure 1:**
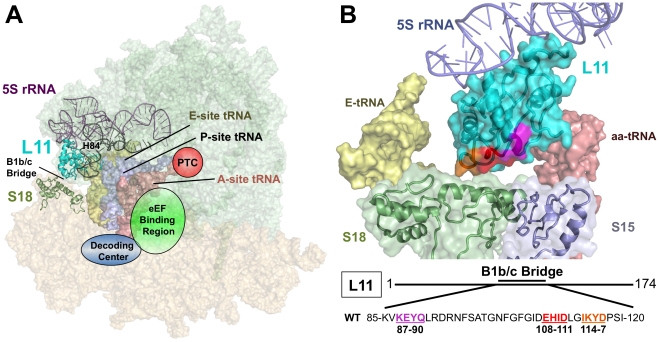
Location of L11 in the ribosome. (**A**) Image of the yeast ribosome. The large subunit is colored green, and the small subunit is pink. L11 (cyan) is located in the central protuberance of the large subunit where it interacts with 5S rRNA, Helix 84 of 25S rRNA, the T-loop of the peptidyl-tRNA, and the small subunit protein S18 via the B1 b and B1 c intersubunit bridges. (**B**) Close-up view of L11 and neighboring structures. Amino acids of L11′s intersubunit region targeted for mutation are colored orange, red, and pink, corresponding to the colored amino acid shown. Ribosomal structures generated in PyMol using yeast cryo-EM [5] fitted with tRNAs from *T. thermophilus* [6].

In the past decade, high resolution X-ray crystallographic and near-atomic resolution cryo-EM datasets have revealed numerous intra- and inter-subunit interactions [1], [2], [3], [4], [5], [6]. In particular, the LSU and SSU directly interact through a series of 17 intersubunit bridges [4], [7]. Most of these involve either RNA-RNA or RNA-protein interactions. An exception is the B1b/c bridge, which is formed between the LSU ribosomal protein L11 in yeast (L5 in bacteria and archaea) and the SSU protein S18 (S13 in bacteria and archaea) [4]. A recent crystal structure of the yeast ribosome identified a second protein-protein intersubunit bridge involving ribosomal protein L3 and a yet to be identified SSU protein bound near h44 and h8, known as the eukaryotic specific eB13 bridge [8]. In yeast, the essential protein L11 is located in the LSU at the intersubunit face of the central protuberance where it interacts with H84 of the 25S rRNA and with 5S rRNA (Figure 1A). L11 can be roughly divided into three regions: the P-site loop (consisting of L11 residues S48-H68) which dynamically interacts with H84 and the P-site tRNA [9], a central region that interacts with the 5S rRNA and H84, and the intersubunit face which participates in the B1b/c intersubunit bridge. Cryo-EM studies of ribosomes sampled through the elongation cycle have revealed a ratcheting motion between the large and small subunits during the process of translocation. This ratcheting breaks the B1 b L11-S18 bridge, resulting in a roughly 30Å movement and adoption of the B1 c bridge conformation [10], [11], [12], [13]. While the ribosome contains numerous intersubunit bridges, the B1b/c bridge undergoes the largest conformational adjustments during the process of ribosomal ratcheting [10], [11], [14]. Previous observations of structural datasets suggested that a series of opposing positive and negative charge motifs between L11 and S18 may provide “sticky and slippery” surfaces that aid in both the movement and placement of the ratcheting subunits [13], [15] (Figure S1).

Previous studies have linked mutations in human L11 to Diamond-Blackfan anemia [16], [17], [18], and L11 has also been shown to play a role in ribosome biogenesis control and regulation of the MDM2-p53 pathway [19], [20], [21]. Further understanding of L11′s role in the ribosome may shed light on the molecular mechanisms underpinning these findings. While it is possible that the B1b/c bridge may merely function to mechanically limit the motions of the ratcheting ribosome, L11′s close proximity to the ribosomal P-site implies it may have an important role in communicating information between different functional centers in the two subunits [9]. The current study focused on identifying the critical amino acid residues of L11 located in the B1b/c bridge region and evaluating their roles in ribosomal function at the biological, biochemical, and structural levels. The observation that disruption of these charges had the greatest impacts on growth and viability is consistent with the view that the differential electrostatic polarities within the B1b/c bridge play a crucial role as a “molecular yardstick” to aid in establishing preferred ratchet orientations [13]. Additionally, we suggest that structural analyses of several mutants has helped to reveal allosteric lines of information transmission in the ribosome, linking the decoding center on the small subunit to the PTC on the large subunit through L11. A model describing L11′s role in a ribosome “wiring diagram” is presented.

## Results

### Generation of *rpL11* alleles and genetic analyses

**Figure 2:**
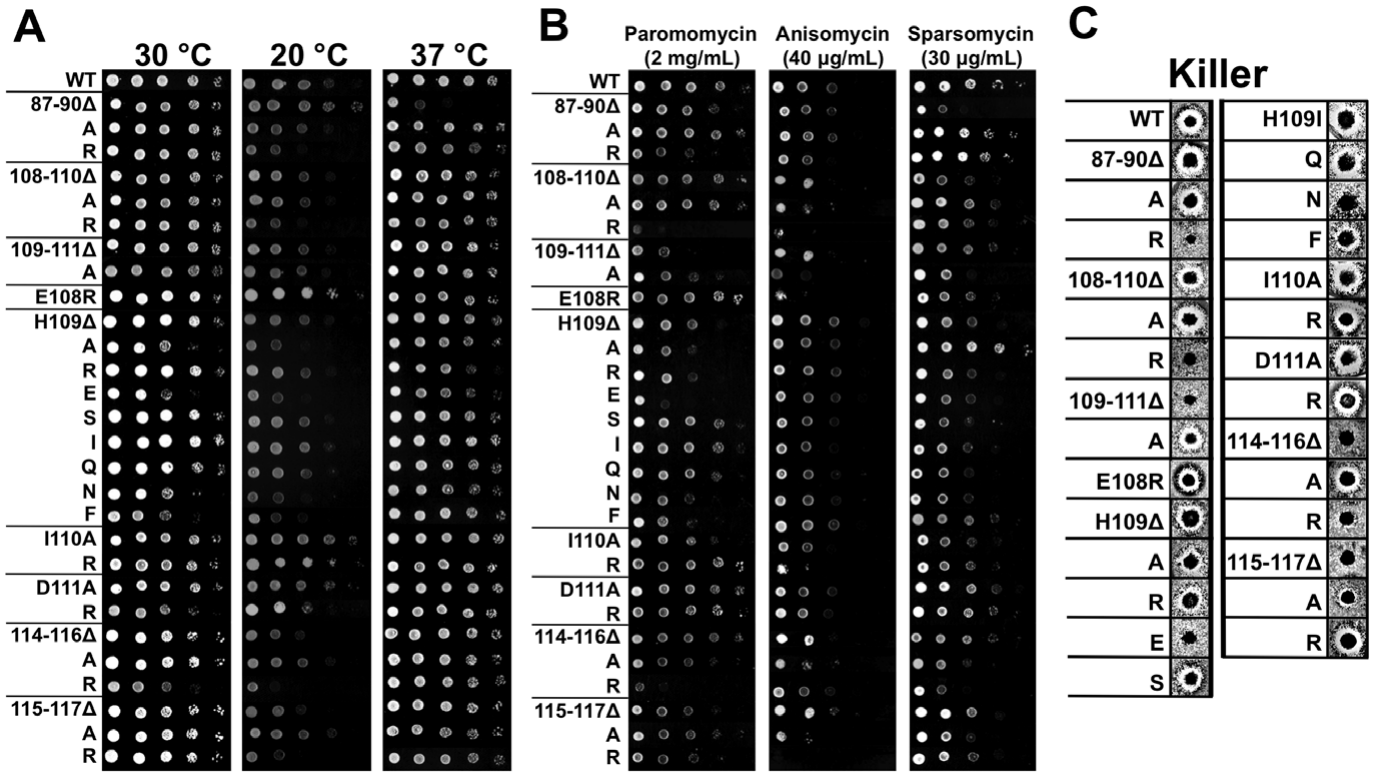
Phenotypic analyses of the viable L11 mutants. (**A**) 10-fold dilutions of indicated yeast strains were spotted onto SD-Trp media and incubated at temperatures indicated, or (**B**) on SD-Trp media containing paromomycin, anisomycin, or sparsomycin at the indicated concentration and grown at 30°C. (**C**) Killer virus assays. Wild-type (WT) Killer^+^ cells are identified by a zone of growth inhibition. Inability to maintain the Killer^+^ phenotype indicates altered translational fidelity.

L11′s essential nature, its close proximity to the tRNA binding sites, and its unusual participation in a protein-protein intersubunit bridge spurred an examination of its role in the relationship between ribosome structure and function, particularly with regard to the transmission of information through the B1b/c intersubunit bridge. Scanning site-directed mutagenesis involving mutation of 3 or 4 sequential amino acids at a time spanning K_87_EYQ_90_, E_108_HID_111_, and I_114_KYD_117_ was initially performed to identify the amino acids of L11 that most affected the contribution of L11 to the B1b/c bridge (Figure 1B). As previous surveys have suggested that movement and positioning of the B1b/c bridge during the intersubunit ratcheting process may be partially controlled by stretches of differentially charged amino acid residues between L11 and S18 [13], [15], each of these three stretches of amino acids were either deleted, changed to alanine, or given a positive charge by mutagenesis to poly-arginine. Of the 15 ‘regional mutants’ created, only H_109_ID_111_ to poly-arginine (109–111R, this nomenclature is used throughout), was unviable. At 30°C, all of the other regional mutants were phenotypically indistinguishable from wild type, except for 114–116R which exhibited a slow growth phenotype (Figure 2A and Table S1).

To determine which amino acids of the lethal mutant were most important for viability, single amino acid alanine and arginine mutations were generated at H109, I110, and D111. Additionally, an H109E mutant was generated to observe the effects of a charge reversal. While I110A and D111A displayed wild-type phenotypes, H109A promoted depressed growth rates at 30°C, and H109E was even further impaired (Figure 2A). For this reason, H109 was selected for further mutagenesis to a wide range of amino acids, as shown in Figure 2A. All H109 mutants were viable. Subsequently, the effects of these mutants on growth rates were monitored by standard 10-fold dilution spot analysis on SD-trp and grown at 30°C, 20°C, and 37°C. Since 30°C represents the optimum temperature for growth of yeast laboratory strains, all other temperature and drug effects were compared to this baseline. At 30°C, slow growth phenotypes were observed in the H109A, H109E, H109N, H109F, D111R, and 114–116R mutants. While several of the regional mutants displayed decreased growth characteristics at 20°C, all of the H109 mutants grew at rates consistent with those observed at 30°C relative to wild-type. At 37°C the slow growth phenotypes observed at 30°C were corrected in the H109A, H109E, H109N, H109F, and D111R mutants. In contrast, growth of H109R was slightly inhibited while that of the 87–90Δ was severely impacted (Figure 2A).

Small molecule translational inhibitors can provide insight into changes in specific functional centers of the ribosome. This study employed 3 such compounds: paromomycin, anisomycin, and sparsomycin. Effects on growth were monitored by dilution spot analysis on SD-trp at the drug concentration indicated in Figure 1B, and cells were grown at 30°C. The aminoglycoside antibiotic paromomycin, which stabilizes near-cognate codon:anti-codon interactions and causes increased rates of translational error [22], was used to probe for effects on the SSU decoding center. Almost all of the regional mutants were sensitive to paromomycin, with especially strong effects seen in the regional arginine mutants, bolstering the hypothesis that charge-charge interactions are important for B1b/c intersubunit bridge function. In the single amino acid H109 mutants, H109Δ, H109E, and H109R were all sensitive to paromomycin, while the remaining mutants were phenotypically comparable to wild-type cells (Figure 2B). Anisomycin binds to the A-site pocket of the LSU, interfering with peptidyl transfer by competing with the 3′ end of the aa-tRNA for binding to the ribosome [23], [24]. Again, sensitivity was observed in the regional mutants, with particularly strong effects observed with the 109–111A, 108–110R, and 115–117R mutants. Conversely, the single amino acid mutants H109A, H109E, H109N, and H109F were all resistant to this drug, with only E108R, H109I, and I110R displaying minor sensitivity (Figure 2B). Sparsomycin binds in conjunction with the peptidyl-tRNA, interfering with peptidyl transfer and aa-tRNA binding, as well as stabilizing hybrid states of the tRNAs [24], [25], [26]. At 30ug/mL of sparsomycin, the 87–90Δ, 108–110Δ, 109–111A, 114–110R regional mutants were hypersensitive as were the H109Δ, H109R, H109S, H109I, and H109Q single amino acid changes. Conversely, the H109A and H109F mutants conferred sparsomycin resistance. This mixture of sparsomycin resistance and sensitivity in the H109 mutants was of particular interest given the previously described interaction of L11 with the peptidyl-tRNA through its “P-site loop” [9].

The yeast “Killer” system can be employed to rapidly screen for general translational fidelity defects. Killer^+^ yeast harbor the L-A and M_1_ dsRNA helper and satellite viruses respectively. The L-A mRNA contains two open reading frames, the first of which encodes the viral capsid protein (Gag), while the second encodes the RNA-dependent RNA polymerase viral replicase (Pol) [27]. Pol is in the −1 reading frame relative to Gag, and a Gag-pol fusion protein is synthesized consequent to a −1 programmed ribosomal frameshift (−1 PRF) [28], [29], [30]. The M_1_ (+) strand RNA is encapsidated and replicated in L-A viral particles, and encodes a secreted toxin that can kill uninfected yeast through its interaction with the Kre1 p cell wall assembly protein [31]. Maintenance of M_1_ is highly sensitive to alterations in translational fidelity [32], and the inability of cells to maintain M_1_ can be scored by loss of the “Killer” phenotype, i.e. loss of a zone of growth inhibition around Killer^+^ cells when plated onto a lawn of diploid Killer^—^ indicator cells. Six of the 14 regional mutants as well as the H109E and H109N mutants exhibited Killer^—^ phenotypes (Figure 2C). Additionally, several of the H109 mutants and the 115–117A regional mutant conferred weak killer phenotypes (Killer^W^) as defined by decreased zones of growth inhibition. The most pronounced effects on Killer virus maintenance were observed in strains in which amino acids were either deleted, or in which the charges of their side-chains were altered. The Killer^+^ phenotype is also sensitive to ribosome biogenesis and subunit joining defects [33]. To determine if loss of Killer could be attributed to this type of defect, polysome and subunit profiles were generated for wild type and Killer^-^ mutant strains by sucrose density fractionation. All of the mutants assayed exhibited wild-type profiles, ruling out subunit joining and biogenesis defects as causative for Killer loss (Figure S2).

### Changes to the B1b/c bridge affect translational fidelity

**Figure 3:**
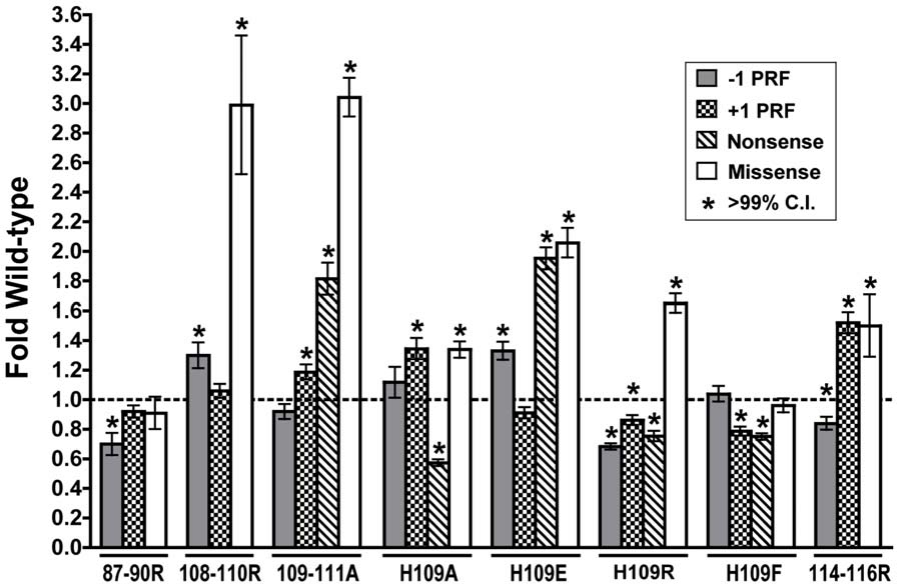
The L11 B1 b/c bridge mutants affect translational fidelity. Isogenic yeast cells expressing either wild-type or mutant forms of L11B were transformed with dual luciferase reporters and control plasmids and rates of translational recoding were determined. All results are graphed as fold wild-type. −1 PRF was measured using the yeast L-A virus frameshift signal. +1 PRF was directed by the frameshift signal derived from the Ty*1* retrotransposable element. Nonsense suppression measures the percentage of ribosomes suppressing an in-frame UAA termination codon positioned between the *Renilla* and firefly luciferase reporter genes. Missense suppression rates evaluated near-cognate utilization of a tRNA^Arg^ tRNA at an AGC serine codon at position 218 within the firefly luciferase gene. Error bars denote standard error. Asterisks above samples indicate statistically significant changes as determined by t-test.

“Translational fidelity” generically refers to the accuracy of protein synthesis. A series of bicistronic *Renilla*-firefly dual luciferase reporters were used to quantifiably examine 4 different aspects of translational fidelity: −1 PRF, +1 PRF, suppression of a nonsense UAA codon, (reviewed in [34]), and utilization of a near-cognate codon (reviewed in [35]). The regional mutant 109–111A plus a subset of the H109 mutants (H109A, H109E, H109R, and H109F) were selected for these analyses based on their locations relative to the lethal 109–111R mutant, and because of their pronounced genetic phenotypes. Rates of −1 PRF were measured using the L-A −1 PRF viral signal positioned between *Renilla* and firefly luciferase genes such that firefly luciferase could only be synthesized consequent to a −1 PRF event. Ratios of fusion luciferase proteins were compared to a 0-Frame control with the luciferase genes in frame with each other. Wild-type rates of −1 PRF were 6.07%±0.16%, i.e. within the 4–8% range observed using other “wild-type” strains in our laboratory of (see [34], [36]). Several mutants conferred statistically significant changes in −1 PRF, including 87–90R, 108–110R, H109E, H109R, and 114–116R (Figure 3).

A recent kinetic analysis of −1 PRF suggests that this phenomenon can occur at three different points during the translation elongation cycle: one occurring when the E- and P-sites are occupied by tRNAs prior to decoding the slippery site, and two occurring while the P- and A-sites contain tRNAs, either during accommodation or peptidyl transfer [37]. In contrast, Ty*1* mediated +1 PRF only occurs when the A-site is empty [38]. Rates of +1 PRF were determined using a *cis*-acting signal obtained from the Ty*1* retrotransposon. In cells expressing wild-type *RPL11B*, +1 PRF rates were 10.98% ±0.30%. L11 mutants 109–111A, H109A, and 114–116R mutants conferred statistically significant increases while H109R, and H109F promoted statistically significant decreases in this measure of translational fidelity (Figure 3).

Codon recognition occurs in the decoding center of the SSU, and is another critical component of translational fidelity. While paromomycin is used as a genetic probe for altered decoding center function, measuring rates of both nonsense codon suppression as well as missense incorporation of near cognate amino acids offers a more precise quantitative analysis of changes in decoding center fidelity. Suppression of a UAA stop codon immediately upstream of the *firefly* luciferase gene was 0.137%±0.003% in cells expressing wild-type L11. All strains tested showed altered rates of nonsense suppression: the 109–111A and H109E mutants all trended higher (∼1.8–2.0 fold increases), while H109A, H109R, and H109F all promoted increased accuracy of UAA decoding. Missense rates were measured by the incorporation of the near cognate tRNA^Arg^ at a mutant AGC serine codon at amino acid 218 of the firefly luciferase reporter, which rescues the activity of this enzyme. Missense rates for wild-type were 0.074% ±0.002, consistent with previous studies [35]. While the 87–90R and H109F mutants promoted wild-type levels of missense incorporation, the remaining mutants all promoted increased rates of missense suppression with 109–111A having the highest at ∼3-fold wild-type (Figure 3). Surprisingly, there did not appear to be a strong correlation between nonsense or missense suppression levels and growth defects induced by paromomycin. While all three tests examine aspects of translational fidelity as it pertains to the decoding center, there are clearly non-overlapping mutational changes affecting each condition, suggesting the decoding center is influenced by the L11 mutants in an indirect or multifaceted manner.

### B1b/c bridge mutants alter tRNA binding

**Figure 4:**
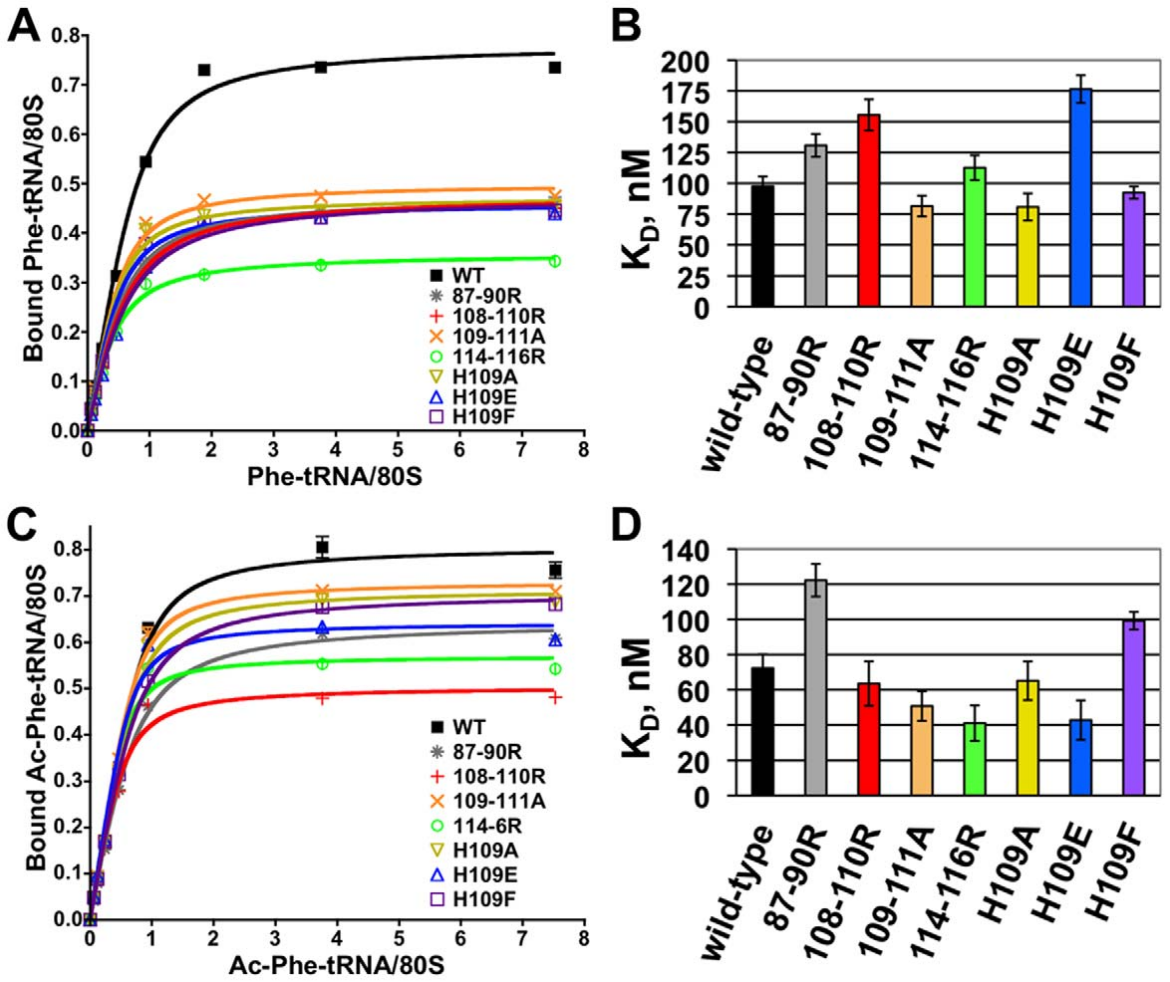
The L11B mutants affect binding affinities for tRNAs. (**A**) Binding of tRNA to the A-site. Ribosomal P-sites were blocked with tRNA^Phe^ at 30°C, then incubated for 35 minutes with [^14^C]Phe-tRNA plus elongation factors and poly(U). 80 S-tRNA-poly(U) complexes were bound to nitrocellulose filters and washed with binding buffer. Samples were read by radioactive scintillation counting. Curves were generated using GraphPad Prism 4. (**B**) A-site tRNA binding K_D_s were determined using one site binding with ligand depletion equation. Error bars depict standard deviation. (**C**) Binding of tRNA to the P-site. Ribosomes were incubated for 40 minutes at 30°C with dilutions of N-acetylated-[^14^C]Phe-tRNA and poly(U) and processed as described for A-site binding. (**D**) K_D_s for P-site tRNA binding. Error bars depict standard deviation.

Several regional arginine and H109 mutants were selected for tRNA binding studies based on their strong genetic phenotypes. Wild-type K_D_ values for A-site tRNA were determined to be 97.6 nM ±12.2 nM. While 109–111A, H109A, H109F, and 114–116R mutant ribosomes did not affect this parameter, ribosomes containing the 87–90R, 108–110R, and H109E mutants of L11 showed decreased affinities for A-site tRNA, with H109E increasing its K_D_ to the greatest extent (176.5 nM ±16.3 nM) (Figures 4A and 4B).

The K_D_ of wild-type ribosomes for Ac-Phe-tRNA^Phe^ in the P-site was 72.3 nM ±7.9 nM. Similar to A-site binding results, the L11 109–111A and H109A mutants, as well as 108–110R did not affect this parameter. Both the 87–90R and H109F L11 mutants conferred higher dissociation constants for P-site tRNA (122.2 nM ±9.3 nM, and 99.2 nM ±5.0 nM respectively), while 114–116R and H109E promoted lower P-site tRNA dissociation constants (41.0 nM ±10.1 and 42.8 nM ±11.2 respectively) (Figures 4C and 4D).

### Ribosome structural conformations are influenced by the B1b/c bridge

**Figure 5:**
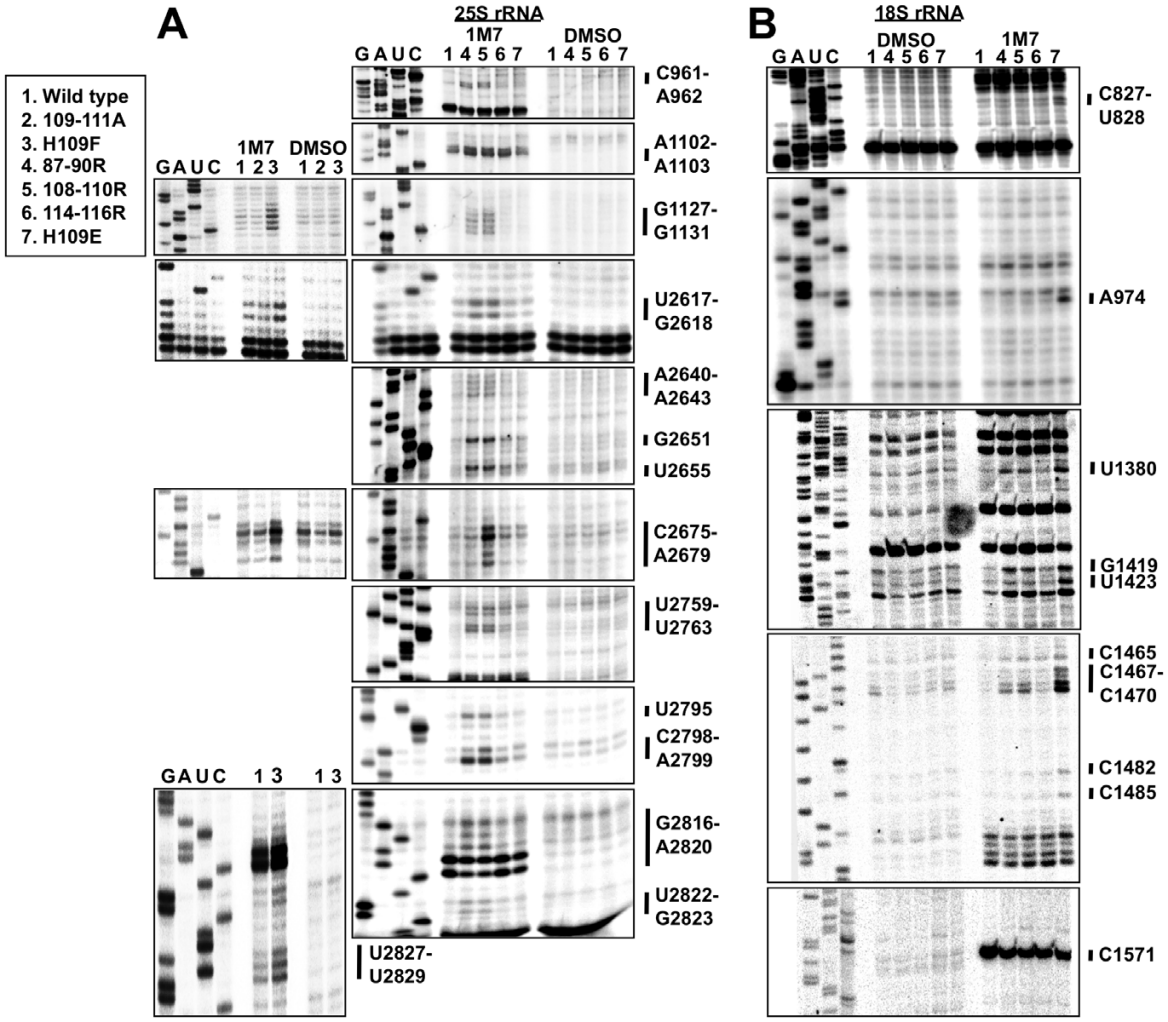
L11 mutants promote local and distant changes in rRNA structure. (**A**)1 M7 SHAPE modification of 25S rRNA for wild-type and mutant puromycin treated salt washed ribosomes. DMSO lanes are unmodified controls. Sequencing ladders are shown to the left of each panel. (**B**) SHAPE modification for 18 S rRNA for same mutant ribosomes. All mutants were probed multiple times, and representative images are presented. Only regions with consistent effects are displayed here.

**Figure 6:**
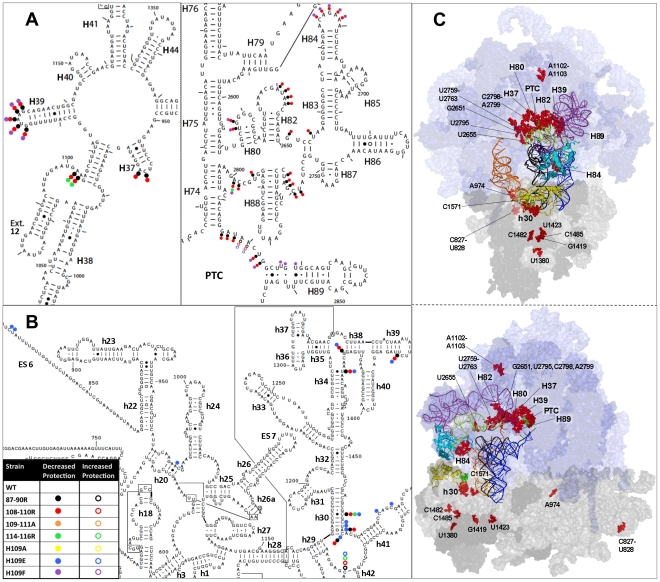
Location of altered rRNA bases. (**A**) Location of altered bases in two dimensional structure of 25S rRNA, and (**B**) in the 18 S rRNA. (**C**) Position of structural changes in three dimensional structure of the ribosome in two separate views. Light blue surface is large subunit rRNA, deeper blue surface are proteins, 5S rRNA is purple, light gray surface is 18 S rRNA, darker gray are proteins. The E, P, and A site tRNAs are orange, black, and deep blue respectively. L11 is in cyan, S18 in yellow, H84 plus extension is lime-yellow. Deprotected bases are shown as red spheres, while bases with increased levels of protection are green spheres.

**S**elective 2′-**h**ydroxyl **a**cylation analyzed by **p**rimer **e**xtension (SHAPE) using the chemical probe 1 M7 [39], [40], [41] was utilized to explore the role of the B1b/c bridge mutants on rRNA conformation and the role of L11 as an informational conduit between distinct regions of the LSU and SSU. SHAPE modification incrementally modifies rRNA base sugar backbones in proportion to their flexibility. Modified (solvent accessible and flexible) bases are visualized as strong stops produced by reverse transcriptase primer extension reactions relative to unmodified samples. In this manner, changes in the levels of flexibility of rRNA bases consequent to L11 mutations can be discerned using SHAPE. As most ribosomal rRNA bases are naturally protected from 1 M7, changes observed in comparing wild-type to mutant ribosomes preferentially appeared as deprotections. In this study, approximately one third of the ribosomal rRNA was interrogated spanning the 5S, 25S, and 18 S rRNAs focusing on those regions closest to L11, the A- and P-site tRNA binding pockets, the PTC, and the decoding center (see Figure S3). Each mutant assayed for tRNA binding was interrogated using SHAPE. The results are shown in Figure 5. Consistent with their lack of effects on tRNA binding, the 109–111A and H109A mutants did not show any structural changes as compared to wild-type ribosomes. While several changes were observed in the H109E and 114–116R mutants, the 87–90R, 108–110R, and H109F mutants conferred numerous changes in the structures of both 25S and 18 S rRNAs, many of which were overlapping. These changes are collectively mapped to both the 2- and 3-dimensional structures in Figure 6 and to individual 3-dimensional mutant ribosomes in Figure S4: all of these bases along with their *E. coli* equivalents are listed in Table S2. Examination of Figure 6 reveals that the majority of changes in rRNA base modifications were concentrated in multiple regions of both the large and small subunit rRNAs. In the large subunit, rRNA structural changes mapped to five distinct regions: (A) nucleotides located in the hairpin loop of Helix 84, which contacts L11 (Figure S5); (B) bases located on top of the LSU tRNA binding pocket including the hairpin loops of Helices 82 and 80, as well as unpaired bases connecting Helix 82 with Helix 83, and flanking Helix 88; (C) the hairpin loop of Helix 39, which interacts with 5S rRNA and ribosomal protein L10; (D) numerous bases located in the core of the PTC centered around A2820 (*E. coli* A2451); (E) the H109F mutant promoted increased reactivity of U2827 – G2829, both of which interact with the N-terminal extension of ribosomal protein L10 [42] (Figure S6). In the small subunit, significant changes in 1 M7 reactivity were observed in 18 S rRNA bases C1465, C1467–C1470, and C1571, which are located between the ribosomal protein S18 binding site [5] and the decoding center (Figure S7). A number of individual bases relatively distantly removed from the mutated B1b/c bridge region also displayed altered protection profiles, including C827, U828, A974, U1380, G1419, U1419, C1482, and C1485.

## Discussion

Cryo-EM analyses show that empty ribosomes, such as were used in the current study, are thermodynamically unconstrained at physiological temperature and can freely assume ∼50 distinct ribosomal conformations [2]. The intersubunit ratcheting process that occurs during translocation results in the most dynamic changes in ribosome structure, with the head of the SSU and the central protuberance of the LSU undergoing the largest conformational changes [10], [11], [13]. The B1b/c bridge, which is formed by L11 and S18, is at the center of this structural rearrangement, and we hypothesize that this bridge may serve as a conduit for the exchange of information among different functional centers in both subunits. The L11 mutants analyzed in the current study are useful not only for testing the hypothesis that the bridge functions as a molecular yardstick by restricting the B1b/c bridge movements to a 30Å conformational adjustment [13], but also to map the allosteric information transmission pathways within the ribosome.

Inspection of Figure 1A reveals that H84 of the LSU is nestled in between two distinct structural elements of L11: the “L11 P-site loop,” which helps the ribosome monitor the tRNA occupancy status of the P-site [9], and the intersubunit bridge region. We recently showed that binding of P-site tRNA to the ribosome reduces the reactivity of H84 as quantified by SHAPE [9]. Local to L11, deprotection of the H84 loop from chemical attack by the 108–110R and H109F mutants, which lie on the opposing side of H84 relative to the L11 P-site loop, suggests that structural changes occurring at the intersubunit B1b/c bridge can shift the dynamic equilibrium of the L11 P-site loop to favor the “P-site empty” state of the ribosome with H84 serving as the intermediary between these two regions of L11 (Figure S5). Thus, the H84 structural changes induced by the mutants assayed in the current study suggest that L11 and H84 work together to communicate information pertaining to the tRNA occupancy status of the P-site and the B1b/c bridge.

While L11 P-site loop mutants only conferred local changes in H84 [9], the B1b/c bridge mutants had wider-ranging effects. H84 forms the distal end of an L-shaped joint, the long axis of which is comprised of Helices 83, 82, and 80. This axis frames the top of the tRNA binding pocket in the LSU from the peptidyl-tRNA T-loop over to the PTC. The observation of numerous changes in the rRNA modification patterns along this axis (Figure S6) suggests that H84 may play a critical role in transmitting information pertaining to the status of the B1b/c bridge to the PTC. However, since the 87–90R mutant caused similar deprotections without affecting H84, deprotection of H84 cannot be the only explanation for the subsequent deprotection of these structures. Importantly, many of the mutants (87–90R, 108–110R and H109F) promoted changes in the hairpin loop of H39. This structure is contacted by ribosomal protein L10 (Figure S8) which has been proposed to play an important role in coordinating tRNA passage through the ribosome [42]. L10 in turn interacts with many different partners, including bases in H89 that are involved in formation of the aa-tRNA accommodation corridor, with the peptidyl-tRNA in the PTC, and with 5S rRNA [5]. Importantly, the chemical protection patterns of A2819 of the PTC and G2828 of H89 were also affected by the Y11C mutant of L10 [42], and G2828 was similarly affected by mutants located in the N-terminal extension region of ribosomal protein L3 [43], thus suggesting a degree of molecular crosstalk between L11 located in the intersubunit face of the central protuberance, and L3/L10 which influence the elongation factor binding site on the LSU and the PTC. Similarly, the protection/deprotection patterns of G2823 and U2827 were affected in ribosomes harboring the C2819U mutant of 25S rRNA located in the PTC [44]. These shared changes in rRNA chemical protection patterns suggest that, while spatially remote, all of these different regions of the ribosome are connected through specific “informational nodes” comprised of specific bases of 25S rRNA.

5S rRNA has also been implicated in information exchange through the LSU [45], [46]. It is tempting to postulate the 5S rRNA may also be involved in transmitting structural signals between these two regions given its position between L11 on one end and the L10-interacting 25S rRNA bases identified here on the other end. While structural evidence for such a mechanism was not detected in the current study, binding of the oligonucleotide used in primer extension analyses and the presence of multiple strong reverse transcriptase stops between nucleotides 1 and 20 limited our interrogation to nucleotides 21–104 of this 121 nucleotide molecule. Additionally, 1 M7 only probes for changes in the ribose sugar, precluding visualization of structural changes affecting the bases themselves. While studies using base-specific chemical probes could potentially address this issue, the presence of a flexible three-way junction in 5S rRNA may enable it to undergo large conformational changes without breakage or formation of new base pairs [47], rendering the detection of changes in 5S rRNA structure opaque to chemical protection methods.

**Figure 7:**
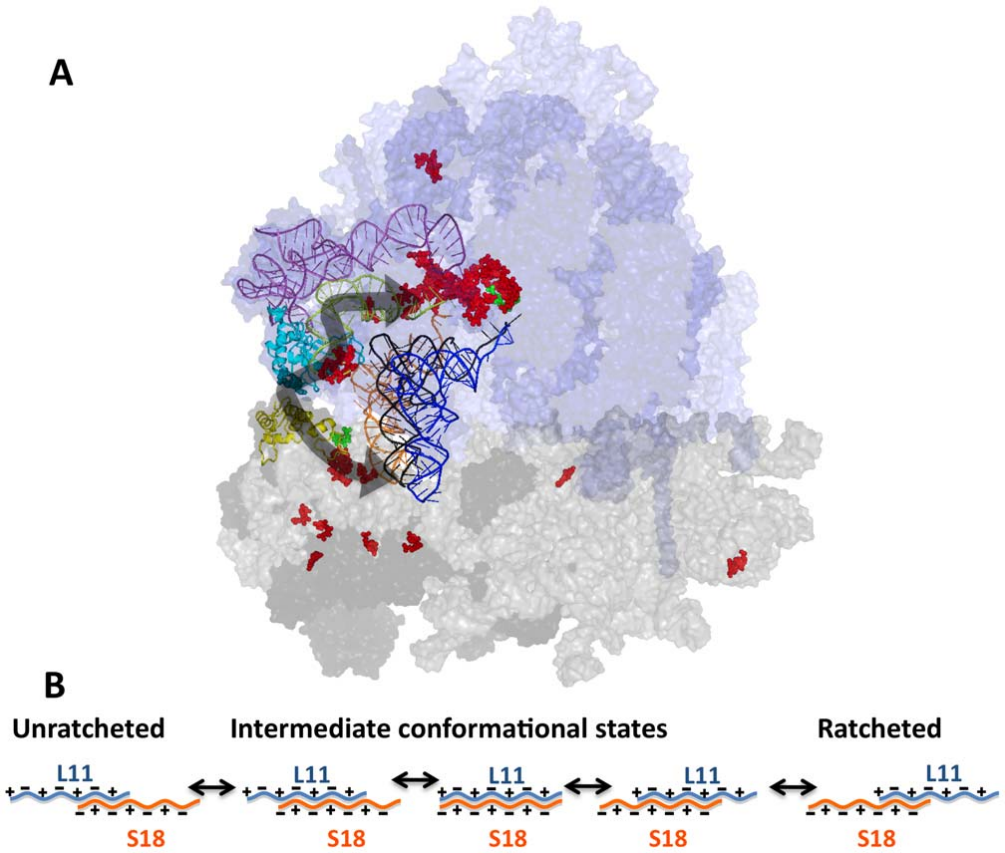
Models describing the B1 b/c bridge and its role in transmitting information between the ribosomal subunits. (**A**) Proposed “wiring diagram.” Overlaid arrows depict communication pathway connecting the decoding center (DC) in the SSU to the PTC in the LSU. Coloration is identical to that used in Figure 6C. (**B**) Cartoon depicting how charge-charge interactions mediate transition of the B1 b/c bridge through an orderly series of allosteric ratcheting states.

Chemical protection analyses also identified a cluster of changes in the 18 S rRNA of the SSU, many of which were concentrated in the region between S18 and the decoding center. These observations indicate that disruptions to the LSU side of the B1b/c bridge have effects on the rRNA structure of the SSU, suggesting this as a pathway for information flow between the decoding center located in the SSU, and functional centers located in the LSU (Figures 6 and 7). These structural changes near the decoding center may also explain some of the paromomycin and translational fidelity effects previously observed. Taken as a whole, rRNA bases affected by the L11 mutants analyzed in this study allow us to begin to map the allosteric information exchange pathways between the major functional centers of both the LSU and the SSU that are linked through the B1b/c bridge. While previous work has focused on the tRNA as a transducer of signaling between the SSU and LSU [48], [49] the structural studies performed in this work were on ribosomes lacking tRNA. This requires alternative pathways to explain the structural modifications visualized. While these observed structural changes might purely reflect an altered preference for ribosomal architectures related to subunit ratcheting, the lack of base changes in the neck (h28) and penultimate stem containing the decoding center (h44) argues for more direct causation of these clustered changes. However, it should be noted (and is discussed below) that other bases further isolated and away from L11 due support the hypothesis that some level of ribosomal global conformation has been affected by the B1 bridge mutations. In summary, we propose that information flows through the B1b/c bridge from the decoding center to the PTC utilizing S18, L11 and H84 across the top of the tRNA binding pocket. This is modeled in Figure 7A.

A significant number of indirect base modification changes were also observed in both ribosomal subunits. These include A1102-A1103 in the LSU, and C827-U828, A974, U1380, G1419, U1423, C1482, and C1485 in the SSU. With no obvious physical connections between these bases or with the B1b/c bridge region, their significance is difficult to ascertain. C827 and U828 are located in the eukaryotic specific expansion segment 6 (ES6). This region of the 18 S rRNA forms an extended helix on the solvent side of the SSU starting below the mRNA decoding platform [8]. ES6 creates two eukaryotic specific intersubunit bridges: eB11 at its tip, in conjunction with ES41 of the LSU, and eB12 at its base, together with protein L19e. This suggests that the changes in C827 and U828 1 M7 protection patterns caused by the L11 mutants were either directly caused by conformation changes in the eB12 bridge, or indirectly by alterations in the eB11 bridge [8]. One explanation for the indirect, long range effects of the L11 B1b/c bridge mutants may be that changes in the conformation of this bridge drive the empty ribosomes used in this study into one or a preferred subset of conformational states. By this model, the changes observed in rRNA structure represent mutant-specific preferences for one or more of the ∼50 conformational states, providing snapshots of the ribosome in intermediate conformations, and bolstering the hypothesis that the opposing electrostatic polar charges of the B1b/c bridge are essential for maintaining the proper equilibrium of the ribosome as a Brownian nano-machine, especially when tRNAs are absent. Consistent with this, the most significant structural changes were observed in the mutations that directly altered the amino acid side-chain charge properties. For example, the only lethal mutant was 109–111R, while 87–90R, 108–110R, 114–116R, and H109E mutants conferred significant changes in rRNA structure. Conversely, the neutral-charged mutations H109A and 109–111A had no discernable effects. This also indicates a significant degree of redundancy built into the intersubunit L11-S18 bridge, as simply voiding a portion of the charge was not sufficient to grossly affect rRNA structure. While structural alterations were observed in H109F, this may be due to the loss of the positively charged histidine replaced by the addition of the bulky phenylalanine side-chain which may inhibit the smooth gliding movement between L11 and S18 during ribosome ratcheting.

Changes in ribosome structure impact its biochemistry. Due to the unique rRNA modification profile of each mutant coupled with multiple regions of overlap, it is not feasible to confidently attribute any one set of rRNA structural changes to the specific effects on tRNA affinities or drug sensitivities. However several trends are worth noting. The 87–90R, 108–110R, and H109E mutants all promoted increased K_D_ values for aa-tRNA, corresponding with modifications in the 18 S rRNA concentrated around the decoding center. H109E conferred the most dramatic structural changes in 18S rRNA, which corresponded with the largest increase in A-site tRNA K_D_. These correlations suggest that structural alterations near the decoding center may disrupt the binding stability of the aa-tRNA. Additionally, these were the only mutants that promoted increased protection of the 25S rRNA bases A2819–A2820 in the PTC, perhaps adding to the instability of the ribosome/aa-tRNA interaction at this site. Similarly, widespread structural changes caused by the 87–90R, H109E, and H109F mutants resulted in altered P-site tRNA dissociation constants. In contrast, both the 109–111A and H109A mutants, which did not have any discernable effects on rRNA structure, did not affect tRNA binding.

Changes in the structure of the ribosome also impact its function. As a gross monitor of translational fidelity, the Killer assay revealed that the L11 mutants that most affected the charge properties of the L11 side of the B1b/c bridge had the greatest effects on Killer virus maintenance. For example, while the 87–90R, 108–110R, and 114–116R mutants were all unable to maintain the Killer virus, their alanine-mutant counterparts all remained Killer^+^. Indeed, while most of the H109 mutants maintained the Killer^+^ phenotype, including H109R which retained a positive charge, H109E was Killer^—^. These observations loosely correlated with changes in −1 PRF, with the Killer^+^ 109–111A, H109A, and H109F mutants all promoting wild-type rates of −1 PRF. In contrast, the 87–90R, 108–110R, H109E, and 114–116R mutants were all Killer^—^ and displayed statistically significant changes in rates of −1 PRF.

A wider examination of translational fidelity revealed numerous mutant-specific effects, i.e. on −1 PRF, +1 PRF, and on the ability of the ribosome to properly identify nonsense and missense codons. Most of the mutants promoted increased rates of missense suppression, which helps stabilize near-cognate codon:anti-codon interactions [50]. In examining the general viability of the mutant yeast strains, slow growth phenotypes observed at the optimal temperature of 30°C were further aggravated by colder temperature, while increased temperature partially corrected this defect for most strains. Lower temperatures decrease entropy, thus decreasing Brownian motion and intermolecular collision rates. In contrast, entropy is greater at higher temperatures, increasing rates of intramolecular unfolding. Thus, the cold-sensitive growth phenotypes observed here suggest that these mutants affected inter-molecular interactions, i.e. interactions between different components of the ribosome, rather than the folding of L11 itself. This is consistent with the widespread changes in rRNA structure discussed above. While the least healthy strains tended to be those affecting amino acid side-chain charge properties, particularly exacerbated by the presence of paromomycin and anisomycin, clearly other strains break these loose rules, and it is likely that other, unknown factors contribute to the overall growth and health of cells. Thus, we propose that the B1b/c bridge region of L11 acts less like a digital “on/off” switch with respect to its role in coordinating intersubunit ratcheting, but rather more like an analog “rheostat,” potentially helping to guide the ribosome through a series of distinct conformational states, depicted in cartoon form in Figure 7B.

In sum, we propose that by tampering with the differentially charged region of L11′s B1b/c intersubunit bridge, we have altered the ability of the ribosomal subunits to fluidly transition between the pre- and post-ratchet states. This may be by increasing the activation energy required to transit between states, or by locking the ribosome in sub-optimal intermediate ratchet states. These structural disruptions had cascading effects on both ribosome biochemistry and on translational fidelity. In disrupting this communication pathway we have identified several distinct regions across the subunits linked with one another through the B1b/c bridge. We have also provided the first experimental evidence, to our knowledge, demonstrating the importance of electrostatic charge interactions in the B1b/c bridge. Future structural analyses of ribosomes arrested at different steps of the translation elongation program will attempt to precisely correlate specific defects in ribosome conformation with translational fidelity and cell growth phenotypes. Additional studies will employ a similar molecular genetics approach to explore this informational pathway from the small subunit side through L11′s protein partner S18 and neighboring S15.

## Materials and Methods

Detailed materials and methods were previously described in a recent publication [9]. Work was conducted in *Saccharomyces cerivisiae* strain JD1313 (*MATα rpl11a::HIS3 rpl11b::HIS3 ura3*–*52 leu2Δ1 trp1Δ63 his3Δ200 Killer^+^ +* YCpL11B URA3) in which YCpL11B URA3 plasmid was replaced with wild type or mutant *RPL11B* flanked by 5′ and 3′ wild-type UTRs episomally supplied to the cell on a TRP1 CEN6 plasmid. Oligonucleotide primers used for generation of L11 bridge mutants are listed in Table S3. The published structures for the 70 S ribosome from *E. coli* (PDB accession numbers: 2AVY, 2AW4; [3], as well as 80 S structures from yeast (3JYV, 3JYW, 3JYX; [5], [51]) were used in the analysis of this work and the generation of figures. Published *Thermus thermophilus* 70 S subunits containing A-site, P-site, and E-site Phe-tRNA were also employed (1G1X [6]). All structures were visualized and manipulated using MacPyMol software [52]. tRNAs were docked into the yeast ribosome using rpS18 as an “anchor” in a tRNAs+S18 object using S18 alignment feature of PyMol. *T. thermophilus* S18 was then hidden for purposes of image generation.

### Temperature and drug resistance/sensitivity assays

Yeast were grown to mid log phase in H–tryptophan synthetic deletion media (-Trp). Strains were set to equivalent OD_595_ values, and cells were serially diluted 10-fold from 10^5^ to 10 CFU per 2.2 µL and spotted on –Trp plates. Growth was monitored at 20°C, 30°C and 37°C, and pharmacogenetic assays utilized 2 mg/ml paromomycin, 40 µg/ml anisomycin, or 30 µg/ml sparsomycin incubated at 30°C for 3–5 days. Killer virus assays were performed as previously described [29].

### Translational Fidelity Assays

The dual luciferase reporter plasmids pYDL-control, pYDL-LA, pYDL-Ty*1*, pYDL-UAA [34] and pYDL-AGC_218_ [35] were utilized to quantitatively measure −1 PRF, +1 PRF, UAA codon readthrough, and suppression of an AGC serine codon in place of an AGA arginine codon in the firefly luciferase catalytic site respectively. The in-frame control was pJD419, the L-A dsRNA virus −1 PRF containing reporter was pJD420, and the Ty*1* containing +1 PRF reporter was pJD421. Rates of nonsense suppression were quantified as previously described [34] using the in-frame control pJD419 and in-frame UAA containing reporter pJD702. The missense reporter plasmid pYDL-AGC containing a firefly luciferase 218 arginine codon (AGA) to serine (AGC) was previously described [34], [35]. Cells were grown in liquid SD media to mid log phase (A_595_ = 0.8–1.5) and lysates were collected. Samples were read in a TD20/20 luminometer using the Stop and Glo dual luciferase kit (Promega). Both control and test lysates were monitored in in triplicate in 6–12 independent experiments per strain depending on the consistency of the data. Frameshifting rates were calculated by taking the ratio of firefly to *Renilla* for control and test reporters, then dividing the average test ratio by the average control ratio to obtain the rates for each recoding event. Results were analyzed using a t-test to determine statistical significance compared to wild-type rates as previously described [36].

### tRNA binding

To monitor binding of aa-tRNA to the A-site, puromycin treated salt washed ribosomes were pre-incubated with soluble binding factors [53], polyuridine, and yeast tRNA^Phe^ to block the P-site, and were subsequently mixed and incubated at 30°C with 2-fold dilutions of yeast [^14^C]Phe-tRNA^Phe^. Complexes were bound to and washed on nitrocellulose filters and tRNA binding was determined by scintillation counting. Dissociation constants were calculated using Graphpad Prism's one site binding with ligand depletion formula. P-site tRNA K_D_s were evaluated in a similar manner with polyU primed puromycin treated salt washed ribosomes incubated with varying concentrations of [^14^C]Ac-Phe-tRNA^Phe^.

### SHAPE structural analysis

Empty puromycin treated salt-washed ribosomes (50 pmol) were resuspended in 200 µl of SHAPE buffer [80 mM Tris-HCl pH 7.4, 100 mM NaCl 15 mM Mg(CH_3_COO)_2_] and incubated for 10 minutes at 30°C. Samples were divided in half and either 10 µl of dimethylsulfoxide (DMSO) or 10 µl of 60 mM 1-methyl-7-nitroisatoic anhydride (1 M7) was added to ribosomes. Samples were incubated at 30°C for 20 minutes. Ribosomes were precipitated, pelletted, and RNA was isolated using an Ambion (Austin, TX) RNAqueous®-Micro RNA isolation kit. Samples were resuspended at a concentration of 1 µg rRNA/7 µl in pure water. HPLC purified oligonucleotide primers purchased from IDT (Coralville, IA) used for structural analysis by SHAPE [41] are listed in Table S4. Oligonucleotides were labeled with γ[^32^P]ATP using T4 polynucleotide kinase (Roche, Indianapolis, IN), and purified from free radiolabeled nucleotides by passage through a MicroSpin G-25 column (GE Healthcare, Piscataway, NJ). Primers were annealed to ribosomal RNA samples and extended using Superscript III enzyme (Invitrogen Life Technologies, Carlsbad, CA). Samples were resolved through 8% urea-acrylamide denaturing gels. Gels were dried and radiolabeled samples were visualized by phosphorimagery. Reported RNA base changes were independently assayed from 2 separate isolations of ribosomal RNA and each reported base was analyzed 2–5 times depending on overall quality of reads (lower quality reads being repeated more) and overlap between adjacent RNA primers. Representative results are provided for each base change in Figure 5.

## Supporting Information

Figure S1 **B1 bridge intersubunit charge motif.** Cartoon view of proteins L11, S18, and S15 with their intersubunit regions shown as surface. Visible are the alternating charges within this region. Coloration: gray is non-polar bases, green are polar, blue are negatively charged, and red are positively charged.
(TIF)

Figure S2 **Polysome and subunit profiles.** Polysomes were generated by 7–47% sucrose gradient fractionation of cycloheximide arrested ribosomes in cell lysate. The absence of halfmer peaks to the right of 80 S and polysome peaks indicated no biogenesis defects caused by the B1 bridge mutants. Subunit profiles were generated in a similar fashion with the omission of cycloheximide and inclusion of 500 mM KCl in the sucrose gradient.
(TIF)

Figure S3 **Three-dimensional representation rRNAs probed by SHAPE.** Locations of 25S, 18S and 5S rRNA bases within the 80S ribosome probed with 1 M7 and their relative proximities to L11. Probed bases are shown in bright purple and viewed from 2 angles.
(TIF)

Figure S4 **Three-dimensional rRNA SHAPE changes for each individual mutant.** Two view angles for each mutant ribosome. rRNA is shown as cartoon. Ribosomal proteins (except for L11 in cyan and S18 in yellow) are omitted from individual mutant diagrams. Coloration is the same as in Figure 6C.
(TIF)

Figure S5 **Position of H84 altered bases relative to B1 bridge and P-site loop.** SHAPE deprotected bases in mutants 108-110R and H109F are shown as red cartoons. L11 mutated amino acids are shown as spheres.
(TIF)

Figure S6 **SHAPE modified bases surrounding the tRNA binding pockets.** Viewed from the top of the large subunit looking down, red sticks depict deprotected bases while green show increased protection from 1 M7 modification. Mutated L11 amino acids shown as teal spheres.
(TIF)

Figure S7 **Clustered SHAPE changes between S18 and decoding center.** 2 views of deprotected (red) and protected (green) bases shown as sticks. Mutated L11 bases shown as teal spheres.
(TIF)

Figure S8 **Position of ribosomal protein L10 relative to nearby modified bases.** Views from two separate angles show L10 and the proximity of its loops to many of the base changes observed in various L11 B1b/c bridge mutants. Red spheres indicate bases with decreased protection/increased flexibility, while green represent increased protection.
(TIF)

Table S1 Summary table of mutant phenotypes.
(DOC)

Table S2 Summary of chemical protection data.
(DOC)

Table S3 Oligonucleotide primers used in the generation of L11 mutants by site directed mutagenesis.
(DOC)

Table S4 Oligonucleotides used for primer extension in SHAPE analyses.
(DOC)

## References

[1] Ban N, Nissen P, Hansen J, Moore PB, Steitz TA (2000). The complete atomic structure of the large ribosomal subunit at 2.4 A resolution.. Science.

[2] Fischer N, Konevega AL, Wintermeyer W, Rodnina MV, Stark H (2010). Ribosome dynamics and tRNA movement by time-resolved electron cryomicroscopy.. Nature.

[3] Schuwirth BS, Borovinskaya MA, Hau CW, Zhang W, Vila-Sanjurjo A (2005). Structures of the bacterial ribosome at 3.5 Å resolution.. Science.

[4] Spahn CM, Beckmann R, Eswar N, Penczek PA, Sali A (2001). Structure of the 80S ribosome from Saccharomyces cerevisiae--tRNA- ribosome and subunit-subunit interactions.. Cell.

[5] Taylor DJ, Devkota B, Huang AD, Topf M, Narayanan E (2009). Comprehensive molecular structure of the eukaryotic ribosome.. Structure.

[6] Yusupov MM, Yusupova GZ, Baucom A, Lieberman K, Earnest TN (2001). Crystal Structure of the Ribosome at 5.5 Å Resolution.. Science.

[7] Gabashvili IS, Agrawal RK, Spahn CM, Grassucci RA, Svergun DI (2000). Solution structure of the E. coli 70S ribosome at 11.5 A resolution.. Cell.

[8] Ben-Shem A, Jenner L, Yusupova G, Yusupov M (2010). Crystal structure of the eukaryotic ribosome.. Science.

[9] Rhodin MHJ, Dinman JD (2010). A flexible loop in yeast ribosomal protein L11 coordinates P-site tRNA binding.. Nucleic Acids Res.

[10] Sergiev PV, Kiparisov SV, Burakovsky DE, Lesnyak DV, Leonov AA (2005). The conserved A-site finger of the 23S rRNA: just one of the intersubunit bridges or a part of the allosteric communication pathway?. Journal of Molecular Biology.

[11] Valle M, Zavialov A, Sengupta J, Rawat U, Ehrenberg M (2003). Locking and unlocking of ribosomal motions.. Cell.

[12] Zhang W, Dunkle JA, Cate JH (2009). Structures of the ribosome in intermediate states of ratcheting.. Science.

[13] Frank J, Gao H, Sengupta J, Gao N, Taylor DJ (2007). The process of mRNA-tRNA translocation.. Proceedings of the National Academy of Sciences of the United States of America.

[14] Frank J, Agrawal RK (2000). A ratchet-like inter-subunit reorganization of the ribosome during translocation.. Nature.

[15] Shasmal M, Chakraborty B, Sengupta J (2010). Intrinsic molecular properties of the protein-protein bridge facilitate ratchet-like motion of the ribosome.. Biochemical and Biophysical Research Communications.

[16] Cmejla R, Cmejlova J, Handrkova H, Petrak J, Petrtylova K (2009). Identification of mutations in the ribosomal protein L5 (RPL5) and ribosomal protein L11 (RPL11) genes in Czech patients with Diamond-Blackfan anemia.. Human Mutation Journal.

[17] Gazda HT, Sheen MR, Vlachos A, Choesmel V, O'Donohue MF (2008). Ribosomal protein L5 and L11 mutations are associated with cleft palate and abnormal thumbs in Diamond-Blackfan anemia patients.. Am J Hum Genet.

[18] Quarello P, Garelli E, Carando A, Brusco A, Calabrese R (2010). Diamond-Blackfan anemia: genotype-phenotype correlations in Italian patients with RPL5 and RPL11 mutations.. Haematologica.

[19] Dai MS, Shi D, Jin Y, Sun XX, Zhang Y (2006). Regulation of the MDM2-p53 pathway by ribosomal protein L11 involves a post-ubiquitination mechanism.. Journal of Biological Chemistry.

[20] Dai MS, Sun XX, Lu H (2010). Ribosomal protein L11 associates with c-Myc at 5 S rRNA and tRNA genes and regulates their expression.. Journal of Biological Chemistry.

[21] Sun XX, Wang YG, Xirodimas DP, Dai MS (2010). Perturbation of 60 S ribosomal biogenesis results in ribosomal protein L5- and L11-dependent p53 activation.. Journal of Biological Chemistry.

[22] Ogle JM, Murphy FV, Tarry MJ, Ramakrishnan V (2002). Selection of tRNA by the Ribosome Requires a Transition from an Open to a Closed Form.. Cell.

[23] Grollman AP (1967). Inhibitors of protein biosynthesis. II. Mode of action of anisomycin.. JBiolChem.

[24] Hansen JL, Moore PB, Steitz TA (2003). Structures of five antibiotics bound at the peptidyl transferase center of the large ribosomal subunit.. JMolBiol.

[25] Schlunzen F, Zarivach R, Harms R, Bashan A, Tocilj A (2001). Structural basis for the interaction of antibiotics with the peptidyl transferase centre in eubacteria.. Nature.

[26] Fredrick K, Noller HF (2003). Catalysis of ribosomal translocation by sparsomycin.. Science.

[27] Icho T, Wickner RB (1988). The MAK11 protein is essential for cell growth and replication of M double-stranded RNA and is apparently a membrane-associated protein.. JBiolChem.

[28] Dinman JD, Icho T, Wickner RB (1991). A -1 ribosomal frameshift in a double-stranded RNA virus forms a Gag-pol fusion protein.. Proc Natl Acad Sci U S A.

[29] Dinman JD, Wickner RB (1992). Ribosomal frameshifting efficiency and Gag/Gag-pol ratio are critical for yeast M1 double-stranded RNA virus propagation.. JVirol.

[30] Wickner RB (1996). Double-stranded RNA viruses of Saccharomyces cerevisiae Microbiol Rev.

[31] Breinig F, Tipper DJ, Schmitt MJ (2002). Kre1p, the plasma membrane receptor for the yeast K1 viral toxin.. Cell.

[32] Dinman JD (1995). Ribosomal frameshifting in yeast viruses.. Yeast.

[33] Ohtake Y, Wickner RB (1995). Yeast virus propagation depends critically on free 60S ribosomal subunit concentration.. Molecular & Cellular Biology.

[34] Harger JW, Dinman JD (2003). An in vivo dual-luciferase assay system for studying translational recoding in the yeast Saccharomyces cerevisiae RNA.

[35] Plant EP, Nguyen P, Russ JR, Pittman YR, Nguyen T (2007). Differentiating between near- and non-cognate codons in Saccharomyces cerevisiae.. PLoS ONE.

[36] Jacobs JL, Dinman JD (2004). Systematic analysis of bicistronic reporter assay data.. Nucleic Acids Res.

[37] Liao PY, Choi YS, Dinman JD, Lee KH (2011). The many paths to frameshifting: kinetic modelling and analysis of the effects of different elongation steps on programmed -1 ribosomal frameshifting.. Nucleic Acids Res.

[38] Clare J, Farabaugh P (1985). Nucleotide sequence of a yeast Ty element: evidence for an unusual mechanism of gene expression.. Proceedings of the National Academy of Sciences of the United States of America.

[39] Merino EJ, Wilkinson KA, Coughlan JL, Weeks KM (2005). RNA structure analysis at single nucleotide resolution by selective 2′-hydroxyl acylation and primer extension (SHAPE).. Journal of the American Chemical Society.

[40] Mortimer SA, Weeks KM (2007). A fast-acting reagent for accurate analysis of RNA secondary and tertiary structure by SHAPE chemistry.. Journal of the American Chemical Society.

[41] Wilkinson KA, Merino EJ, Weeks KM (2006). Selective 2′-hydroxyl acylation analyzed by primer extension (SHAPE): quantitative RNA structure analysis at single nucleotide resolution.. Nat Protoc.

[42] Petrov AN, Meskauskas A, Roshwalb SC, Dinman JD (2008). Yeast ribosomal protein L10 helps coordinate tRNA movement through the large subunit.. Nucleic Acids Res.

[43] Meskauskas A, Dinman JD (2008). Ribosomal protein L3 functions as a ‘rocker switch’ to aid in coordinating of large subunit-associated functions in eukaryotes and Archaea.. Nucleic Acids Res.

[44] Rakauskaite R, Dinman JD (2008). rRNA mutants in the yeast peptidyltransferase center reveal allosteric information networks and mechanisms of drug resistance.. Nucleic Acids Res.

[45] Dinman JD (2005). 5S rRNA: Structure and Function from Head to Toe.. International Journal of Biomedical Science.

[46] Kiparisov S, Petrov A, Meskauskas A, Sergiev PV, Dontsova OA (2005). Structural and functional analysis of 5S rRNA.. Molecular Genetics and Genomics.

[47] Besseova I, Reblova K, Leontis NB, Sponer J (2010). Molecular dynamics simulations suggest that RNA three-way junctions can act as flexible RNA structural elements in the ribosome.. Nucleic Acids Research.

[48] Cochella L, Green R (2005). An active role for tRNA in decoding beyond codon:anticodon pairing.. Science.

[49] Rodnina MV, Gromadski KB, Kothe U, Wieden HJ (2005). Recognition and selection of tRNA in translation.. FEBS Letters.

[50] Carter AP, Clemons WM, Brodersen DE, Morgan-Warren RJ, Wimberly BT (2000). Functional insights from the structure of the 30S ribosomal subunit and its interactions with antibiotics.. Nature.

[51] Spahn CM, Gomez-Lorenzo MG, Grassucci RA, Jorgensen R, Andersen GR (2004). Domain movements of elongation factor eEF2 and the eukaryotic 80S ribosome facilitate tRNA translocation.. EMBO J.

[52] DeLano WL (2006). The PyMOL Molecular Graphics System, version 1.4.. http://www.pymol.org/.

[53] Dresios J, Derkatch IL, Liebman SW, Synetos D (2000). Yeast ribosomal protein L24 affects the kinetics of protein synthesis and ribosomal protein L39 improves translational accuracy, while mutants lacking both remain viable.. Biochemistry.

