# Supplementary figures and images for: An Extensive Network of Information Flow through the B1b/c Intersubunit Bridge of the Yeast Ribosome
Source: PLoS One. 2011 May 19;6(5):e20048. doi: 10.1371/journal.pone.0020048 (PMC3098278; doi:10.1371/journal.pone.0020048)

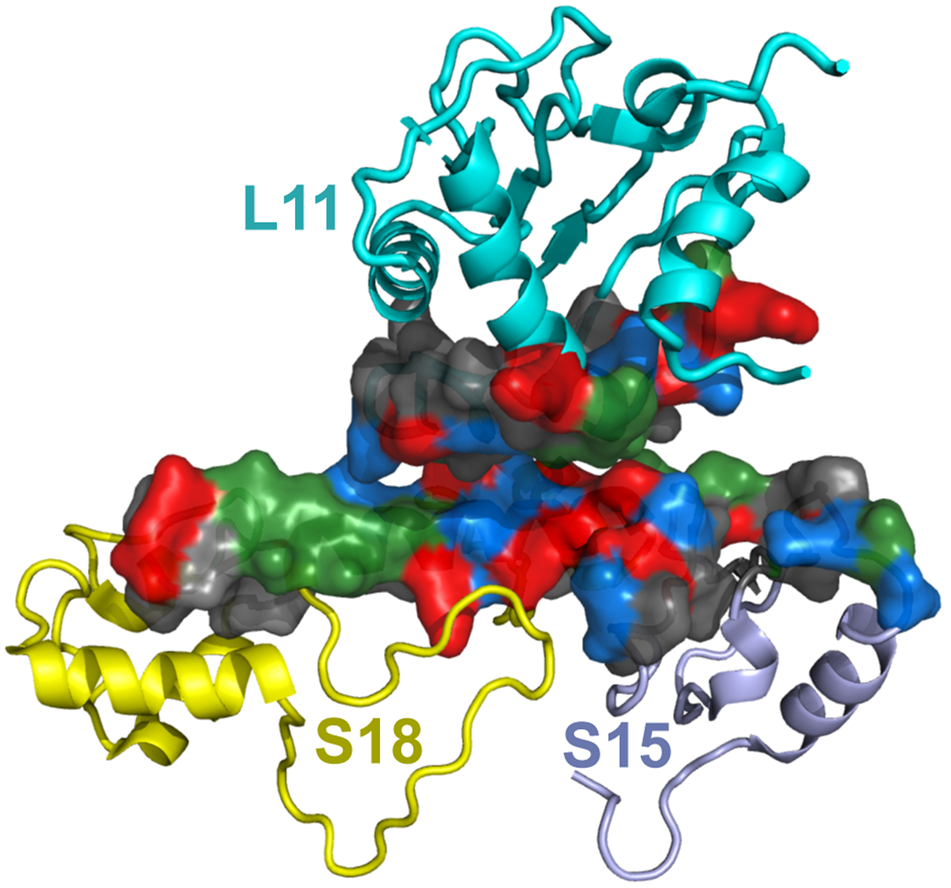

Supplement: Figure S1 — B1 bridge intersubunit charge motif. Cartoon view of proteins L11, S18, and S15 with their intersubunit regions shown as surface. Visible are the alternating charges within this region. Coloration: gray is non-polar bases, green are polar, blue are negatively charged, and red are positively charged. (TIF) [file pone.0020048.s001.tif]

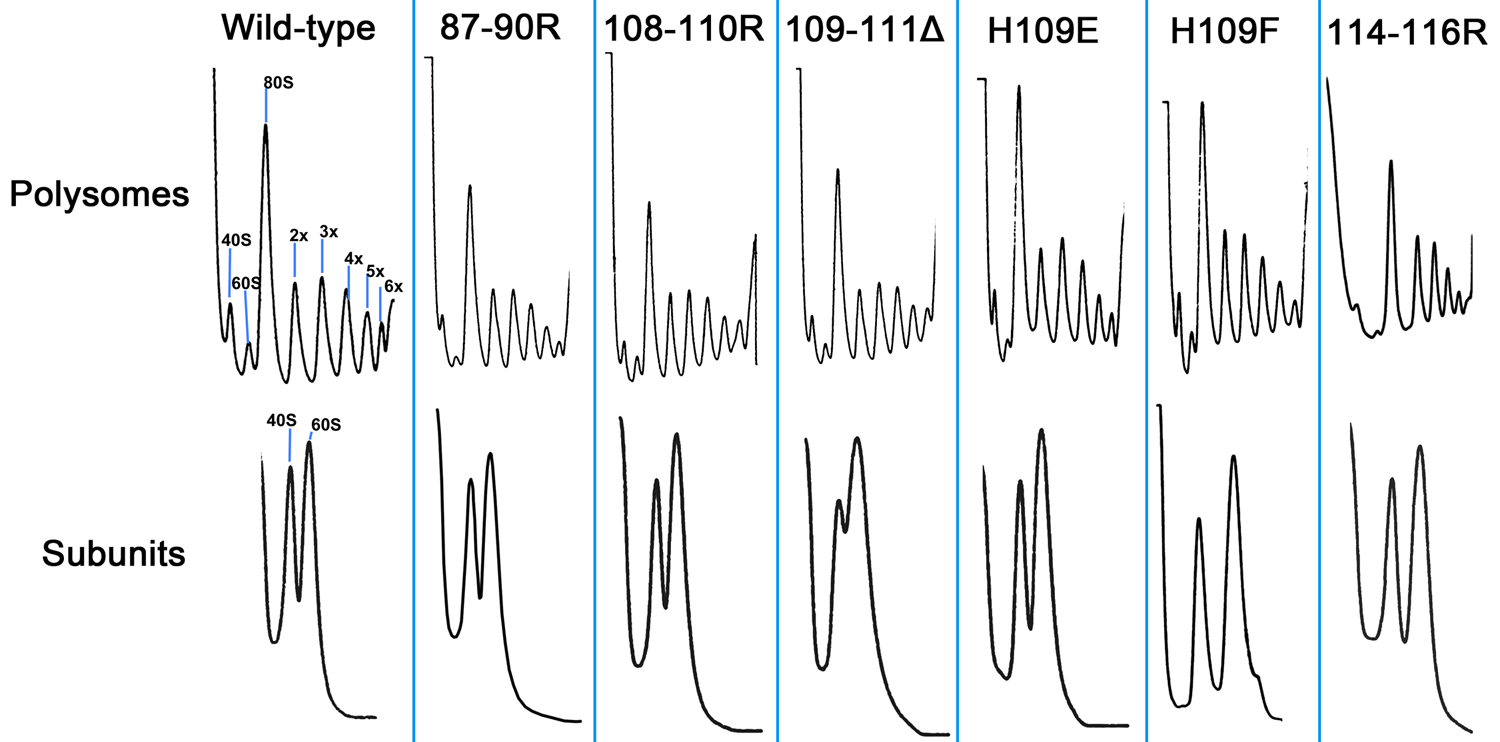

Supplement: Figure S2 — Polysome and subunit profiles. Polysomes were generated by 7–47% sucrose gradient fractionation of cycloheximide arrested ribosomes in cell lysate. The absence of halfmer peaks to the right of 80 S and polysome peaks indicated no biogenesis defects caused by the B1 bridge mutants. Subunit profiles were generated in a similar fashion with the omission of cycloheximide and inclusion of 500 mM KCl in the sucrose gradient. (TIF) [file pone.0020048.s002.tif]

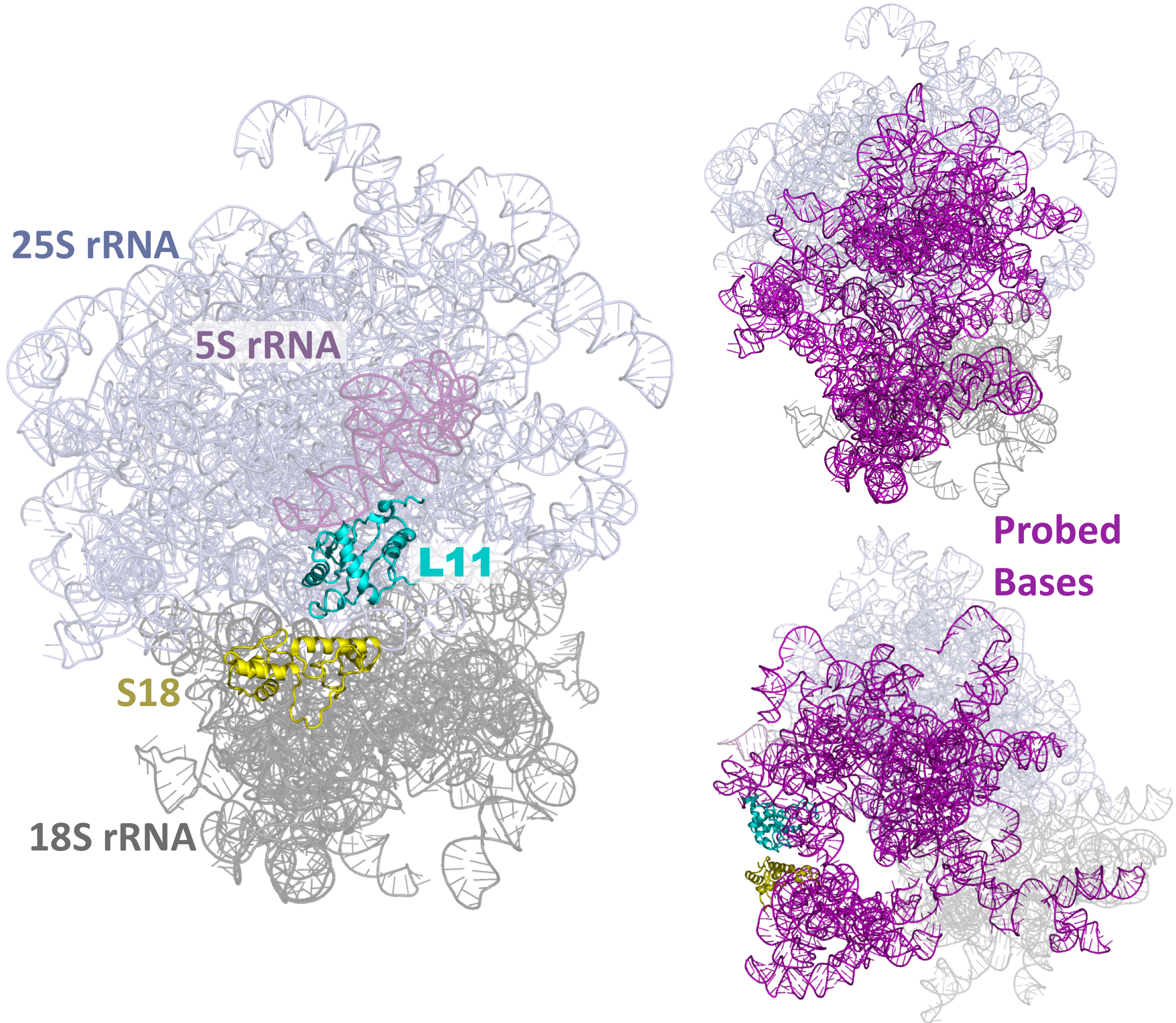

Supplement: Figure S3 — Three-dimensional representation rRNAs probed by SHAPE. Locations of 25S, 18S and 5S rRNA bases within the 80S ribosome probed with 1 M7 and their relative proximities to L11. Probed bases are shown in bright purple and viewed from 2 angles. (TIF) [file pone.0020048.s003.tif]

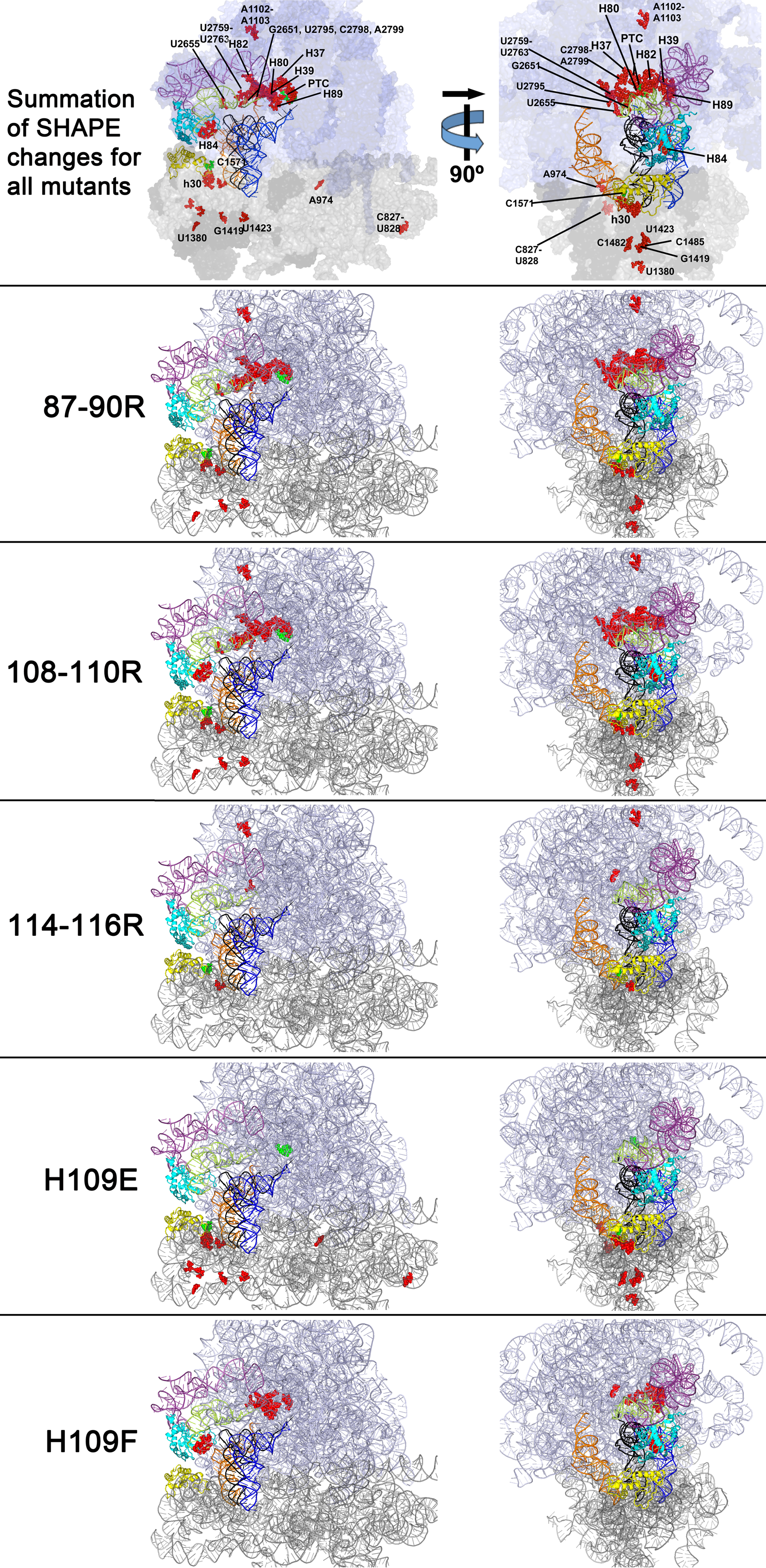

Supplement: Figure S4 — Three-dimensional rRNA SHAPE changes for each individual mutant. Two view angles for each mutant ribosome. rRNA is shown as cartoon. Ribosomal proteins (except for L11 in cyan and S18 in yellow) are omitted from individual mutant diagrams. Coloration is the same as in Figure 6C. (TIF) [file pone.0020048.s004.tif]

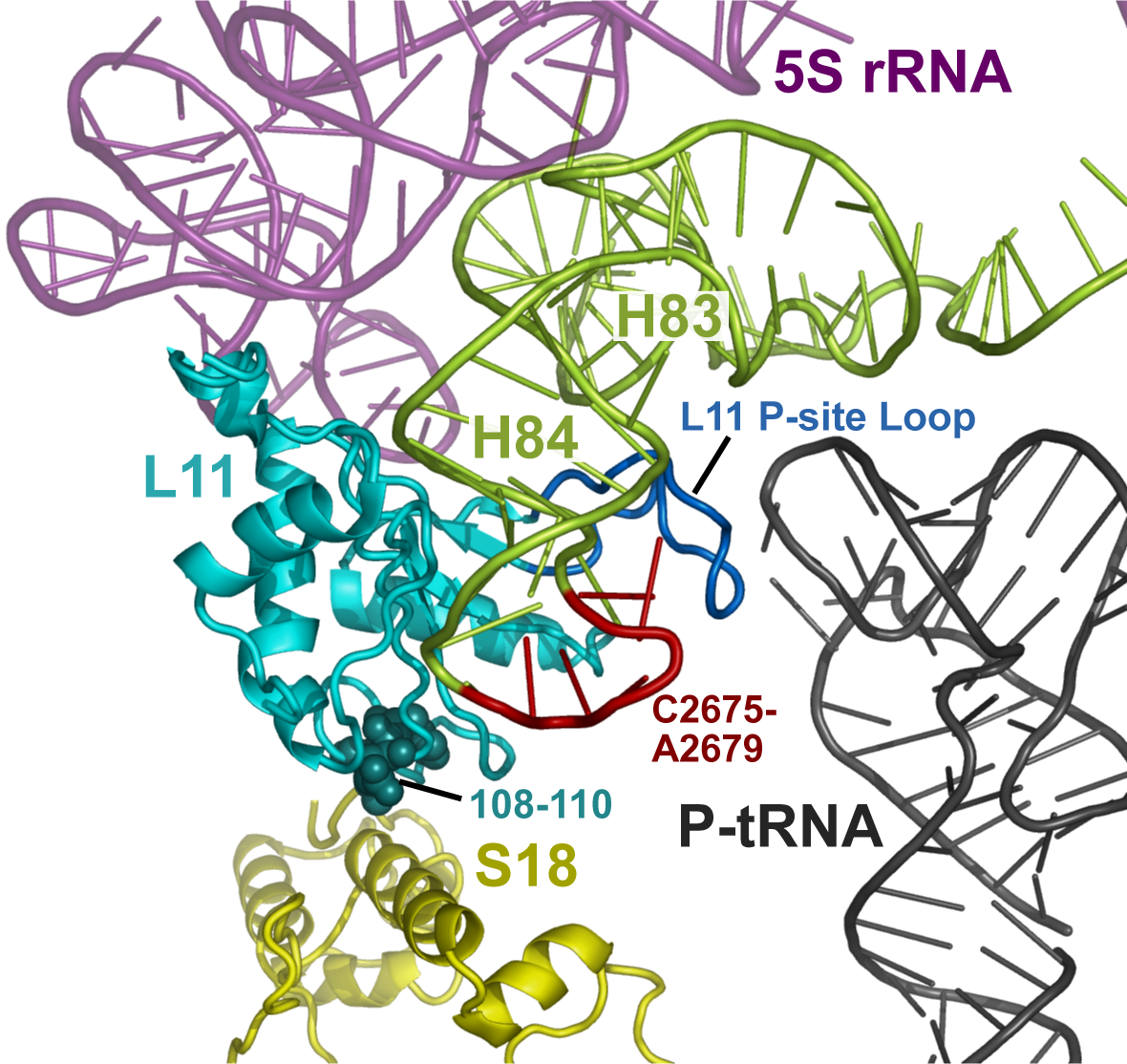

Supplement: Figure S5 — Position of H84 altered bases relative to B1 bridge and P-site loop. SHAPE deprotected bases in mutants 108-110R and H109F are shown as red cartoons. L11 mutated amino acids are shown as spheres. (TIF) [file pone.0020048.s005.tif]

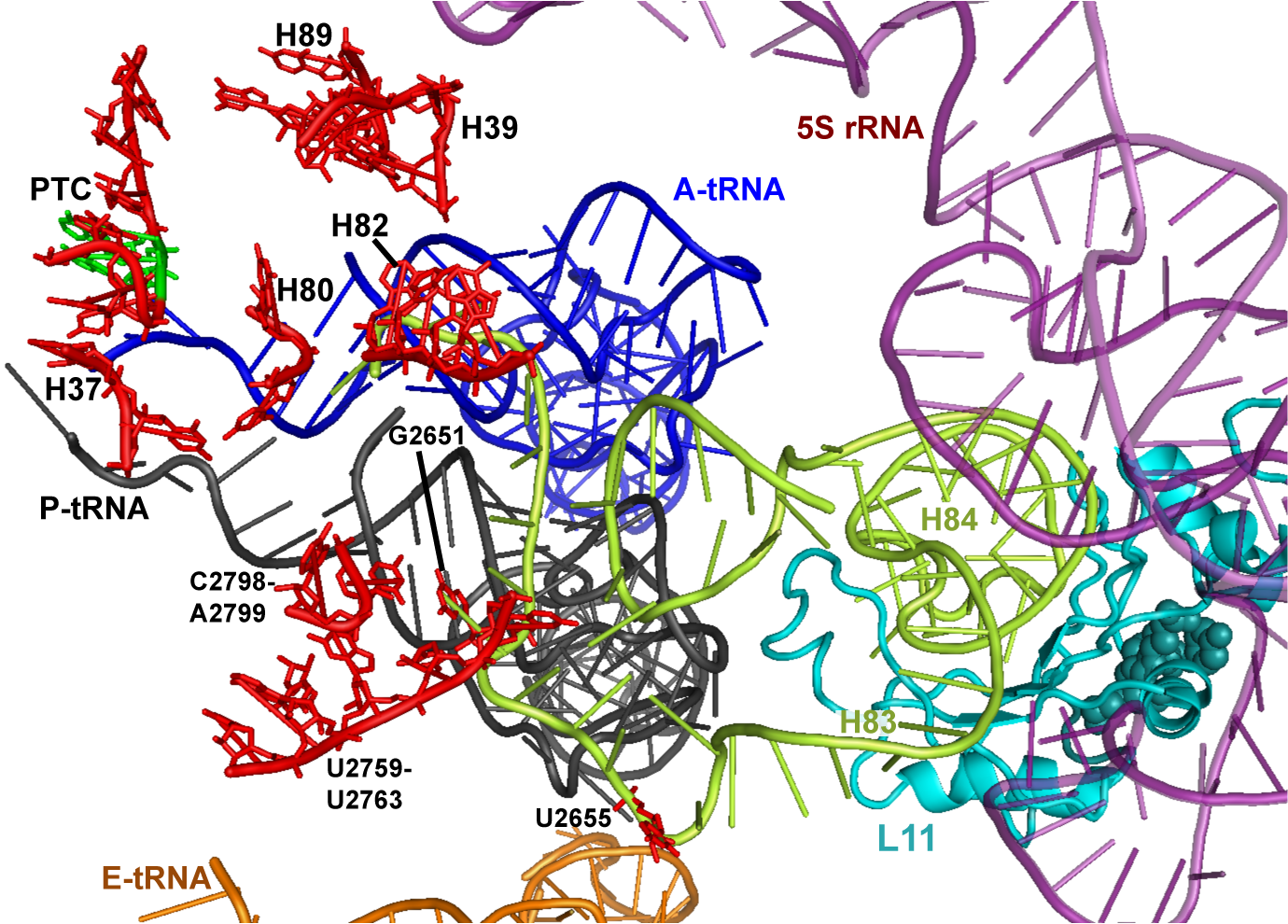

Supplement: Figure S6 — SHAPE modified bases surrounding the tRNA binding pockets. Viewed from the top of the large subunit looking down, red sticks depict deprotected bases while green show increased protection from 1 M7 modification. Mutated L11 amino acids shown as teal spheres. (TIF) [file pone.0020048.s006.tif]

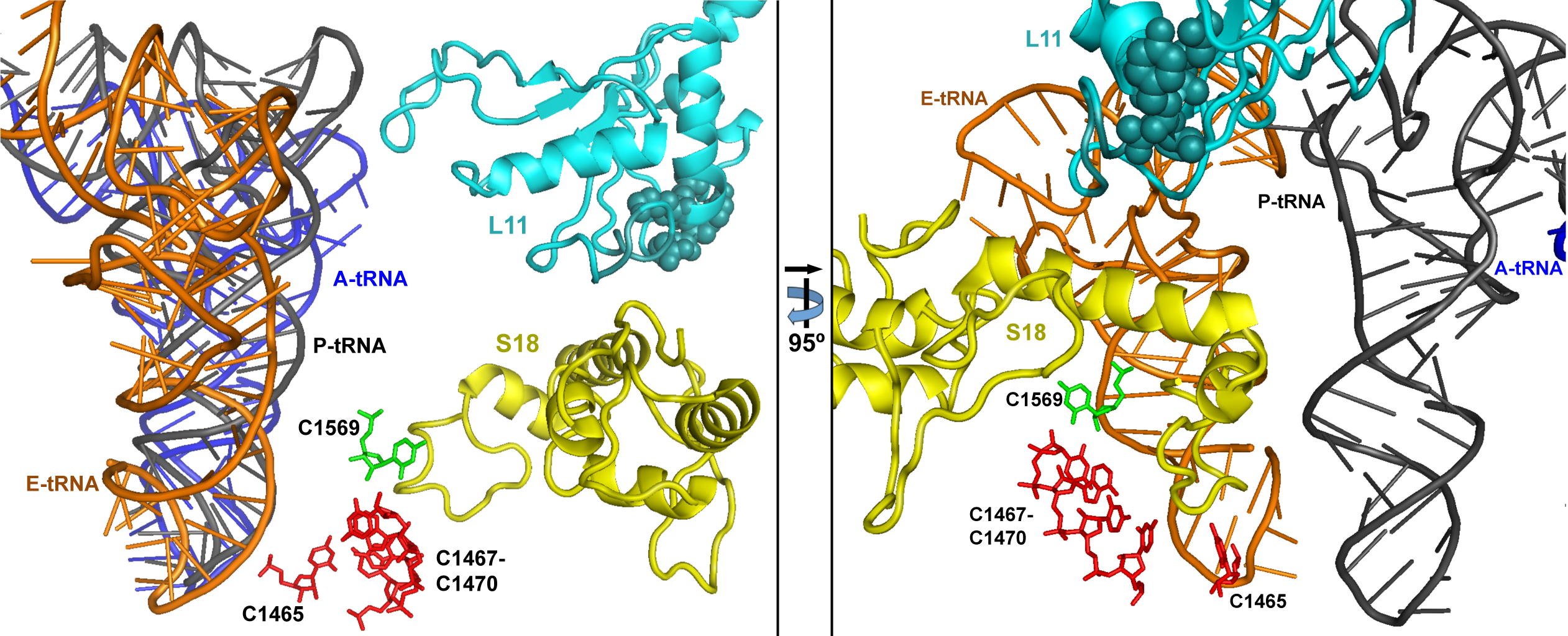

Supplement: Figure S7 — Clustered SHAPE changes between S18 and decoding center. 2 views of deprotected (red) and protected (green) bases shown as sticks. Mutated L11 bases shown as teal spheres. (TIF) [file pone.0020048.s007.tif]

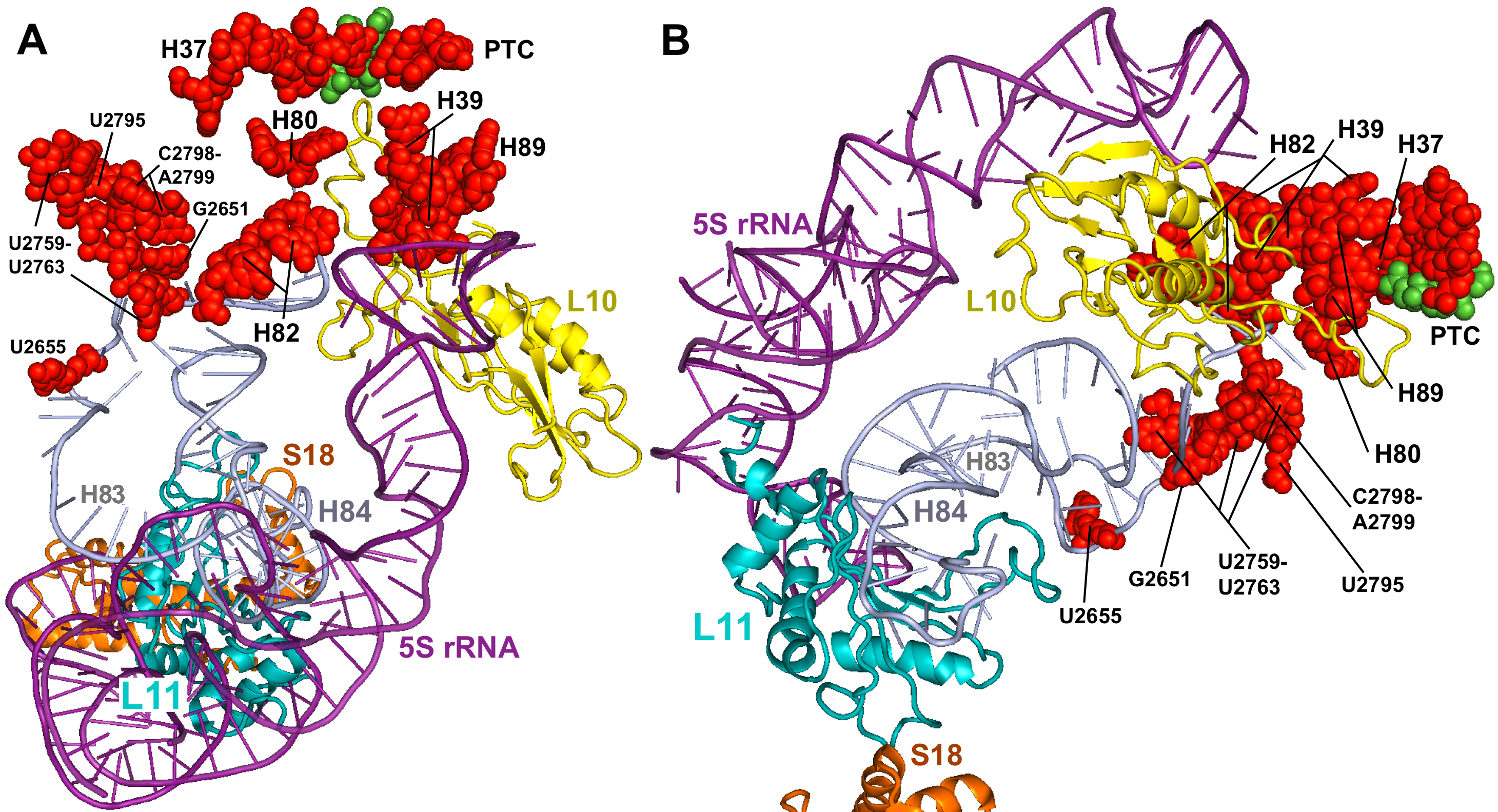

Supplement: Figure S8 — Position of ribosomal protein L10 relative to nearby modified bases. Views from two separate angles show L10 and the proximity of its loops to many of the base changes observed in various L11 B1b/c bridge mutants. Red spheres indicate bases with decreased protection/increased flexibility, while green represent increased protection. (TIF) [file pone.0020048.s008.tif]

**Table S2.** Summary of chemical protection data.


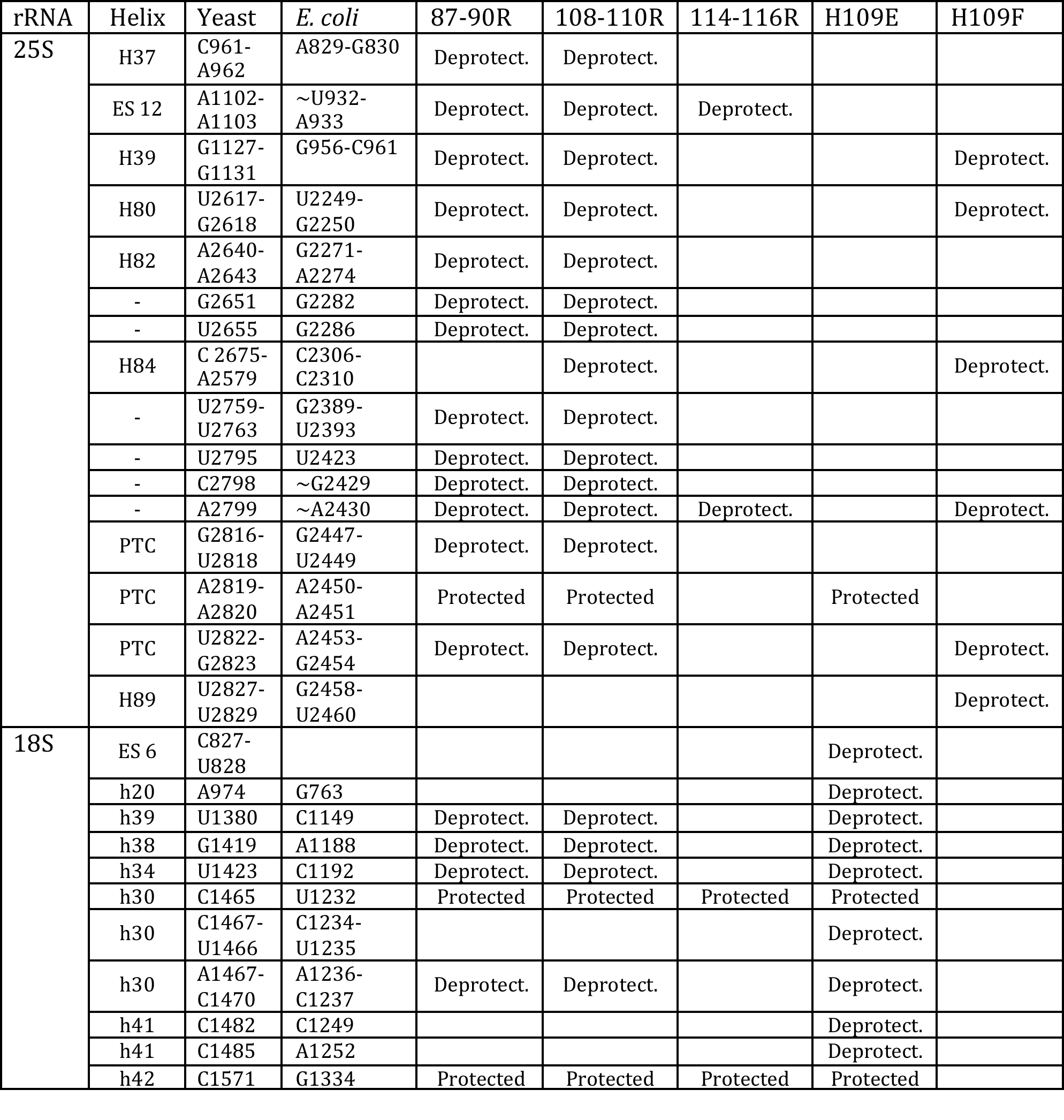

Supplement: Table S2 — Summary of chemical protection data. (DOC) [file pone.0020048.s010.doc]

**Table S4.** Oligonucleotides used for primer extension in SHAPE analyses.


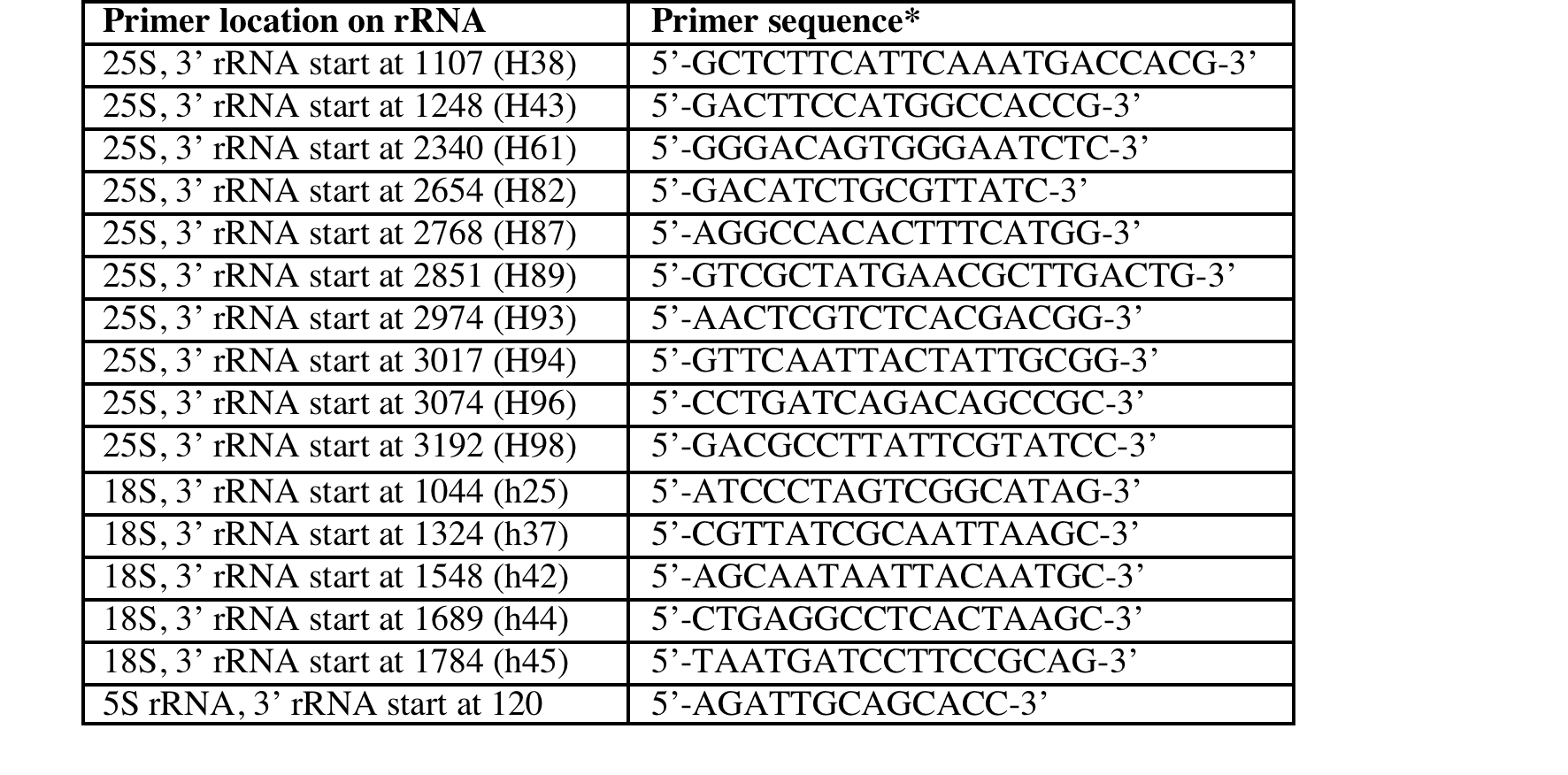

Supplement: Table S4 — Oligonucleotides used for primer extension in SHAPE analyses. (DOC) [file pone.0020048.s012.doc]
