# Supplementary material for: An Extensive Network of Information Flow through the B1b/c Intersubunit Bridge of the Yeast Ribosome
Source: PLoS One. 2011 May 19;6(5):e20048. doi: 10.1371/journal.pone.0020048 (PMC3098278; doi:10.1371/journal.pone.0020048)
Supplement: Table S1 — Summary table of mutant phenotypes. (DOC) [file pone.0020048.s009.doc]

**Table S1.** L11 Mutant Phenotype Summary

| **L11 Mutant** | **Viability** | **30°C Growth** | **20°C Growth** | **37°C Growth** | **Paromomycin** | **Anisomycin** | **Sparsomycin** | **Killer** | **-1 PRF** | **+1 PRF** | **Nonsense** | **Missense** | **A-tRNA K_D_** | **P-tRNA K_D_** | **SHAPE** |
| --- | --- | --- | --- | --- | --- | --- | --- | --- | --- | --- | --- | --- | --- | --- | --- |
| 87-90Δ | yes | wt | resistant | hyper sensitive | sensitive | wt | hyper sensitive | wt | - | - | - | - | - | - | - |
| 87-90A | yes | wt | wt | wt | wt | wt | wt | wt | - | - | - | - | - | - | - |
| 87-90R | yes | wt | sensitive | wt | sensitive | wt | wt | loss | decrease | wt | - | wt | increase | increase | changes |
| 108-110Δ | yes | wt | wt | wt | wt | wt | sensitive | wt | - | - | - | - | - | - | - |
| 108-110A | yes | wt | wt | wt | wt | wt | sensitive | wt | - | - | - | - | - | - | - |
| 108-110R | yes | wt | sensitive | wt | hyper sensitive | sensitive | sensitive | loss | increase | wt | - | increase | increase | wt | changes |
| 109-111Δ | yes | wt | wt | wt | sensitive | wt | sensitive | loss | - | - | - | - | - | - | - |
| 109-111A | yes | wt | wt | wt | sensitive | sensitive | sensitive | wt | wt | increase | increase | increase | wt | wt | wt |
| 109-111R | no | - | - | - | - | - | - | - | - | - | - | - | - | - | - |
| E108R | yes | wt | resistant | wt | resistant | sensitive | sensitive | wt | - | - | - | - | - | - | - |
| H109Δ | yes | wt | wt | wt | sensitive | wt | sensitive | wt | decrease | wt | increase | increase | - | - | - |
| H109A | yes | sensitive | wt | resistant | wt | resistant | resistant | wt | wt | increase | decrease | increase | wt | wt | wt |
| H109R | yes | wt | wt | sensitive | sensitive | wt | sensitive | wt | decrease | decrease | decrease | increase | - | - | - |
| H109E | yes | sensitive | wt | resistant | sensitive | resistant | wt | loss | increase | wt | increase | increase | increase | decrease | changes |
| H109S | yes | wt | wt | wt | wt | wt | sensitive | wt | - | - | - | - | - | - | - |
| H109I | yes | wt | wt | wt | wt | wt | sensitive | wt | - | - | - | - | - | - | - |
| H109Q | yes | wt | wt | wt | wt | wt | sensitive | wt | - | - | - | - | - | - | - |
| H109N | yes | sensitive | wt | resistant | wt | wt | wt | loss | - | - | - | - | - | - | - |
| H109F | yes | sensitive | wt | resistant | wt | resistant | resistant | weak | wt | decrease | decrease | wt | wt | increase | changes |
| I110A | yes | wt | resistant | wt | sensitive | wt | sensitive | wt | - | - | - | - | - | - | - |
| I110R | yes | wt | resistant | wt | resistant | sensitive | sensitive | wt | - | - | - | - | - | - | - |
| D111A | yes | wt | resistant | wt | resistant | wt | wt | wt | - | - | - | - | - | - | - |
| D111R | yes | sensitive | wt | resistant | resistant | wt | resistant | wt | - | - | - | - | - | - | - |
| 114-116Δ | yes | wt | sensitive | wt | resistant | wt | wt | loss | - | - | - | - | - | - | - |
| 114-116A | yes | wt | wt | wt | sensitive | wt | sensitive | wt | - | - | - | - | - | - | - |
| 114-116R | yes | sensitive | wt | resistant | sensitive | wt | sensitive | loss | decrease | increase | - | increase | wt | decrease | changes |
| 115-117Δ | yes | wt | sensitive | wt | sensitive | wt | sensitive | loss | - | - | - | - | - | - | - |
| 115-117A | yes | wt | wt | wt | wt | sensitive | sensitive | weak | - | - | - | - | - | - | - |
| 115-117R | yes | wt | sensitive | wt | sensitive | sensitive | sensitive | wt | - | - | - | - | - | - | - |
