# Supplementary material for: An Extensive Network of Information Flow through the B1b/c Intersubunit Bridge of the Yeast Ribosome
Source: PLoS One. 2011 May 19;6(5):e20048. doi: 10.1371/journal.pone.0020048 (PMC3098278; doi:10.1371/journal.pone.0020048)

**Table S3.** Oligonucleotide primers used in the generation of L11 mutants by site directed mutagenesis.


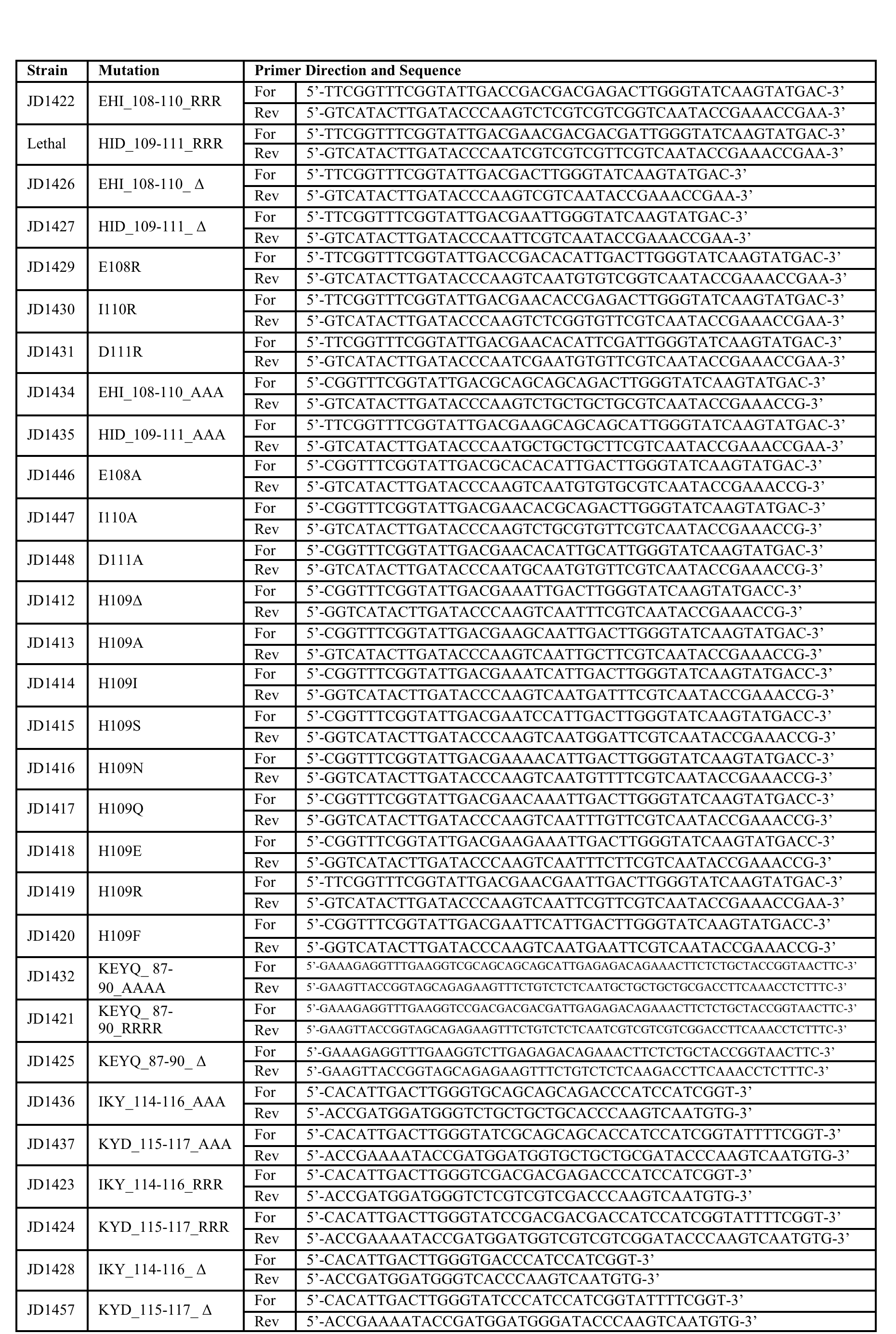

Supplement: Table S3 — Oligonucleotide primers used in the generation of L11 mutants by site directed mutagenesis. (DOC) [file pone.0020048.s011.doc]
